# Region-based analysis of sensory processing using diffusion tensor imaging

**DOI:** 10.1371/journal.pone.0284250

**Published:** 2023-04-10

**Authors:** Kai Nakagawa, Yongjeon Cheong, Seonkyoung Lee, Kaie Habata, Taku Kamiya, Daichi Shiotsu, Ichiro M. Omori, Hidehiko Okazawa, Hirotaka Kosaka, Minyoung Jung

**Affiliations:** 1 Department of Neuropsychiatry, University of Fukui, Eiheiji, Japan; 2 Cognitive Science Research Group, Korea Brain Research Institute, Daegu, Republic of Korea; 3 Biomedical Imaging Research Center, University of Fukui, Eiheiji, Japan; 4 Research Center for Child Mental Development, University of Fukui, Eiheiji, Fukui, Japan; 5 Division of Developmental Higher Brain Functions, Department of Child Development, United Graduate School of Child Development, University of Fukui, Eiheiji, Fukui, Japan; Cook Children\’s Health Care System: Cook Children’s Medical Center, UNITED STATES

## Abstract

The caudate nucleus has been thought to be involved in the control of motor commands by the cerebrum, and recent studies suggest that it may play a role in the control of attachment behavior, cognition, emotion, and mental functions. Implied by the basal ganglia’s involvement in the execution, planning and control of movement, the caudate nucleus functions in a situation-dependent manner where processing of external stimuli is important on the basis of learning and memory. Sensory processing, which determines the response to external stimuli, has been shown to be related to various brain regions but it remains unknown how sensory processing is associated with the structure of the caudate nucleus and white matter microstructures of the caudate. Using four diffusion parameters derived from diffusion tensor imaging (DTI) (i.e., fractional anisotropy (FA), mean diffusivity (MD), axonal diffusivity (AD), and radial diffusivity (RD)) and the Adolescent/Adult Sensory Profile (AASP) questionnaire of 99 healthy subjects [42 males and 57 females; mean age:26.9 years, standard deviation 6.9], we investigated the relationship between white matter structure in the caudate nucleus and sensory processing. In consistent with what had been suggested by the results of previous studies, we found significant correlations between AD, MD and tactile sensation. Furthermore, we found a significant correlation between AD, MD and tactile sensory avoidance, the AASP sub-scores regarding the tactile senses. To the best of our knowledge, this is the first study to show that DTI diffusion parameters correlate with AASP scores in specific brain regions.

## Introduction

Sensory processing is a physiological response to the upcoming sensory events triggered by stimuli encountered in the environment [1]. Well-organized sensory processing helps social interaction during daily life. For example, well-organized visual sensory processing integrates visual stimuli, such as gaze, facial expressions, and biological movements, to help rich social interaction, which in turn affects daily living skills and ultimately quality of life [2–4]. In contrast, abnormal sensory processing is highly likely to interfere with the acquisition of essential daily living skills and social interaction during infancy and early childhood [5].

To understand sensory processing in daily life, Dunn et al. proposed a theory of sensory processing to explain the diversity of responses to individual behavior [6]. According to Dunn’s theory behavioral responses to sensory stimuli are classified into two states: sensory sensitivity and low sensory registration. The behavioral responses to sensory stimuli are simultaneously categorized whether they are active and passive depending on the degree of avoidance of the response to the sensory stimulus [7]. Following Dunn’s theory, the Sensory Profile questionnaire was developed to quantify individual variabilities in sensory processing with respect to tendencies of behavioral responses to various sensory stimuli such as visual, auditory, touch, taste/smell, movement, and activity level [8]. In addition, a previous study has shown that sensory profiles can be useful in assisting in the identification of individual changes in sensory processing [9]. Although sensory profiles are significantly useful in assessing changes in sensory processing in individuals, the use of questionnaires for this purpose raises the difficulty of self-monitoring. In the case of subjects with low intelligence, they may not be able to understand the meaning of the questions when the sensory profile is based on the questionnaire. In addition, previous studies have reported a lack of self-monitoring even in subjects with normal intelligence levels [10]. To improve our understanding of human sensory processing, biological evidence is needed to assess human sensory processing, regardless of intelligence level and limitation of questionnaires.

At this stage, we do not have an objective method of assessing sensory processing, but neuroimaging studies indicate that the caudate nucleus has the potential to objectively assess sensory processing [11–13]. For instance, caudate nucleus connectivity, including other brain regions, has been associated with tactile sensory processing [14] and sensory sensitivity [12]. The caudate nucleus is connected directly or indirectly by white matter through the cerebral cortex or thalamus, and it is also known to be involved in social interaction and sensory processing [15]. However, sensory processing depends not only on the caudate nucleus but also on the white matter that adversely affects the integration of sensory information and sensory processing in the sensory-related central nervous network, which is positioned as an input site for the caudate nucleus [16]. Looking further at the correlation between the caudate nucleus and white matter, it has been shown that changes in the white matter structure of the caudate nucleus correlate with blood flow in the caudate nucleus cortex [17]. To date, no study has investigated white matter in the caudate nucleus related to sensory processing.

To assess human sensory processing with white matter structure, we used diffusion tensor imaging (DTI), which is based on diffusion-weighted images and evaluates white matter microstructure based on the diffusion of water molecules in nerve fibers. DTI is a significantly useful method for the analysis of white matter microstructure, and the number of studies using DTI has been increasing in recent years [18]. DTI-based assessment of white matter microstructure has four different parameters: fractional anisotropy (FA), mean diffusivity (MD), axonal diffusivity (AD), and radial diffusivity (RD). FA is the total magnitude of water directional movement along the axonal fiber, and a sensitive indicator of changes in white matter structure. MD is a mean of all three axes of the diffusion ellipsoid and reflects the rate of water diffusion within a voxel, independent of the directionality, AD describes the mean diffusion coefficient of water molecules diffusing parallel to the tract, RD can be defined as the magnitude of water diffusion perpendicular to the tract, and these all reflect the integrity of white matter structures [18–20]. This study aimed to elucidate the correlation between the white matter structure of the caudate nucleus and sensory processing. We analyzed the white matter structure of the caudate nucleus by DTI, and sensory processing was assessed using the Japanese version of the Adolescent/Adult Sensory Profile (AASP).

## Materials and methods

### Participants

Typically developed individuals were recruited via internet posts around Fukui Prefecture in Japan. The exclusion criteria were a history of brain injury, history of major medical conditions, or left-handedness. Experienced psychiatrists (I.M.O. and H.K.) administered a short-structured diagnostic interview using the Mini-International Neuropsychiatric Interview [21] to exclude psychiatric disorders, psychiatric symptoms, excessive alcohol consumption, or problems with medication. This study was conducted in accordance with the Health Insurance Portability and Accountability Act guidelines, and informed consent was obtained from all participants. The study was also approved by the Ethics Committee at the University of Fukui (approve# 20170182).

### Adolescent/Adult Sensory Profile (AASP)

The Japanese version of the AASP was used to assess the sensory processing [22]. The AASP is a standardized self-report questionnaire with a total of 60 items and a 5-point Likert scale. The AASP consists of sensory processing modalities and quadrants, visual, auditory, tactile, olfactory, gustatory, and motor, and contains six modalities of activity levels, with 10, 11, 13, 8, 8, and 10 items, respectively. The total score of the items in each style is calculated, and the higher the total score, the stronger the atypical tendency of the corresponding sensory processing. In addition, the AASP has four quadrant scores: “low registration,” “sensation seeking,” “sensory sensitivity,” and “sensation avoiding.” Each of these four quadrant scores consists of 15 items. These four quadrant states are based on Dunn’s sensory processing model [22], which categorizes the tendency to respond to sensory stimuli in terms of neurological thresholds and behavioral responses. Low registration refers to high neurological thresholds and a tendency to exhibit passive behavioral responses, which implies sensory dullness and a tendency to be unaware of even strong sensory stimuli. Sensation-seeking behavior requires specific sensory stimuli to meet high neurological thresholds and stabilize oneself. Sensory sensitivity refers to the tendency to have low neurological thresholds due to passive behavioral responses and to feel pain when exposed to strong or even mild sensory stimuli. Sensory avoidance refers to the tendency to avoid sensory stimuli with low neurological thresholds and hatred through active behavioral responses. Information on the participants’ scores (mean, SD, range, and possible score) for each style and quadrant is shown in Table 1.

**Table 1:** Demographic characteristics.

Values	Mean	Standard deviation	Range	Possible score
Number(N)	99			
(men/women)	(42/57)			
Age	26.9	6.9	19–43	
BMI	21.8	2.7	17.7–30.3	
Adolescent/Adult Sensory Profile				
*Modality-specific subscale*				
Visual	23.2	4.7	11–35	10–50
Auditory	22.6	5.3	12–39	11–55
Touch	29.2	6.3	15–48	13–65
Taste/Smell	16.8	3.6	8–25	8–40
Movement	17.3	3.6	9–26	8–40
Activity level	26.4	4.7	14–37	10–50
*Quadrant scores*				
Low registration	27.8	6.6	15–48	15–75
Sensation seeking	40.3	7.0	23–55	15–75
Sensory sensitivity	33.9	7.1	19–51	15–75
Sensation avoiding	33.5	7.3	17–49	15–75

### Magnetic resonance imaging acquisition

Magnetic resonance (MR) images were acquired using a 3-Tesla positron emission tomography (PET)/MR scanner (SIGNA PET/MR; General Electric Medical Systems, Milwaukee, WI, USA) with an 8-channel head coil. High-resolution T1-weighted anatomical MR imaging (MRI) and DTI were acquired using the scan parameters of our previous study [23]. The detailed scan parameters were as follows: T1-weighted anatomical MRI (repetition time = 6.38 ms, echo time [TE] = 1.99 ms, flip angle = 11°, field of view = 256 mm, number of slices = 172, voxel dimension = 1.0 mm × 1.0 mm × 1.0 mm) and DTI (single-shot echo-planar imaging, acquisition matrix = 128 × 128, TE = minimum, repetition time = 9327 ms, field of view = 240 mm, pixel size = 1.9 mm^2^ × 1.9 mm^2^, number of axial slices = 45, slice thickness/gap = 3.0 mm/0 mm, 30 distributed isotropic orientations, b-values of 1000 s/mm^2^ and 0).

### Diffusion tensor imaging analysis

DTI images were rated for quality using a 6-point Likert scale (unusable, poor, fair, good, very good, and excellent) by an experienced researcher (M.J., K.N., or H.O.) and excluded if an image was rated as unusable, poor, or fair poor MR and/or diffusion image quality based on previous studies [24, 25]. DTI was performed using DSI studio software [26]. The fiber tracking algorithm was used, and all fiber bundles were performed using the Runge-Kutta algorithm, which was initially set for all imaging [27]. The FA threshold was randomly selected, the angular threshold was randomly selected between 15° and 90°, and the step size was randomly selected between 0.5 voxel and 1.5 voxel. A total of 5,000 seed points were placed. The trajectory of the fibers was smoothed by averaging the propagation direction using 30% of the leading direction. Length of nerve bundles < 30 mm or more than 300 mm were excluded. The end region that is white matter tracts that are terminated at the caudate nucleus were determined and the diffusion parameters (FA, MD, RD, and AD) of the white matter structure were extracted.

### Statistical analysis

We performed partial correlation analysis (Statistical Package for the Social Sciences, version 20; IBM Corporation) to evaluate the strength of the correlation between AASP scores (six modality-specific scores, four quadrant scores) and diffusion measures (FA, MD, AD, and RD) of caudate nucleus white matter by considering age and sex-specific differences as control variables. Additionally, to examine AASP in further detail, we performed partial correlation analysis to evaluate the correlation between the sub-scores of AASP (24 items) and diffusion parameters of each white matter pathway using age and sex differences as control variables. The details of the AASP scores and sub-scores are shown in Tables 1 and 2. In all partial correlation analyses, a p-value of <0.0006 was considered statistically significant.

**Table 2:** Demographic characteristic of Adolescent/Adult Sensory Profile sub-scores.

Values	Mean	Standard deviation	Range	Possible score
Low registration-Taste/Smell	3.1	1.2	2–7	2–10
Sensation seeking-Taste/Smell	7.0	2.0	3–12	3–15
Sensory sensitivity-Taste/Smell	2.4	1.3	1–5	1–5
Sensation avoiding-Taste/Smell	4.3	1.7	2–10	2–10
Low registration-Movement	3.3	1.2	2–6	2–10
Sensation seeking-Movement	6.9	2.4	2–10	2–10
Sensory sensitivity-Movement	5.8	2.0	3–12	3–15
Sensation avoiding-Movement	1.3	0.7	1–4	1–5
Low registration-Visual	3.6	1.3	2–8	2–10
Sensation seeking-Visual	5.6	1.8	2–10	2–10
Sensory sensitivity-Visual	6.9	2.3	3–13	3–15
Sensation avoiding-Visual	7.0	2.1	3–13	3–15
Low registration-Touch	4.9	1.8	3–12	3–15
Sensation seeking-Touch	7.8	2.3	3–14	3–15
Sensory sensitivity-Touch	9.2	2.8	4–17	4–20
Sensation avoiding-Touch	7.4	2.7	3–15	3–15
Low registration-Activity level	6.9	2.4	3–15	3–15
Sensation seeking-Activity level	7.8	1.9	3–12	3–15
Sensory sensitivity-Activity level	3.2	1.1	1–5	1–5
Sensation avoiding-Activity level	8.5	2.1	3–13	3–15
Low registration-Auditory	5.9	2.0	3–12	3–15
Sensation seeking-Auditory	5.1	1.9	2–10	2–10
Sensory sensitivity-Auditory	6.4	1.9	3–11	3–15
Sensation avoiding-Auditory	5.1	2.0	3–12	3–15

## Results

### Participant demographic information

The sample in the final analysis comprised 99 young adults with typical development (42 males and 57 females; mean age: 26.9 years, standard deviation [SD]: 6.9). To test the normal distribution of AASP in our sample, we used the Kolmogorov-Smirnov test. The AASP scores in our sample had a normal distribution (p>0.05). The details of AASP scores are presented in Tables 1 and 2.

### Correlations between AASP scores and diffusion parameters in the caudate nucleus

**Fig 1:**
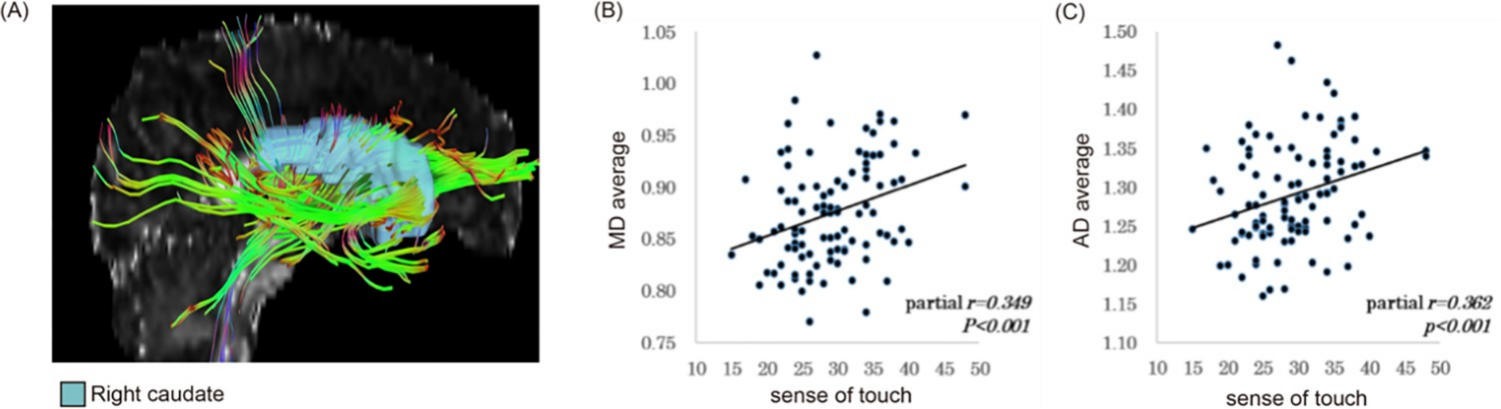
Correlation between the white matter fiber of the right caudate and AASP modality scores. A shows white matter in the right caudate using DSI studio. B shows the correlations between MD of the white matter in the right caudate and the sense of touch score in AASP score (partial r = 0.349, p<0.001). C shows the correlations between AD of the white matter in the right caudate and the sense of touch score in AASP score (partial r = 0.362, p<0.001). AD, axonal diffusivity; MD, mean diffusivity; AASP, Adolescent/Adult Sensory Profile.

To examine the relation between the diffusion characteristics of the white matter microstructure in the bilateral caudate nuclei and sensory processing, we performed a series of partial correlation analyses of diffusion parameters and AASP scores in the bilateral caudate nuclei with age and sex as control variables. While no significant correlation appeared between AASP scores and the diffusion parameters of left caudate nucleus, we found significant correlations for the right caudate nucleus as follows: correlation between MD and touch scores of AASP (partial r = 0.349, p<0.001) and correlation between AD and touch scores of AASP (partial r = 0.362, p<0.001). The results of the correlation analysis are presented in Table 3 and Fig 1.

**Table 3:** Association between diffusion parameters and Adolescent/Adult Sensory Profile (AASP) scores or AASP sub-scores.

Seed region	Diffusion tensor index	AASP scores	Partial correlation coefficient	*p value*
		*Modality*		
Right caudate	MD	Sense of touch	0.349	<0.0006
	AD	Sense of touch	0.362	<0.0006
		*sub-score*		
Right caudate	AD	Sensation avoiding-Touch	0.367	<0.0003
	MD	Sensation avoiding-Touch	0.365	<0.0003

AD, axonal diffusivity; MD, mean diffusivity; AASP, Adolescent/Adult Sensory Profile

To further examine sensory processing in more detail, we subdivided the style-specific items according to quadrant. For example, for item “I get distracted when there is loud noise around me,” the modality-quadrant was set as “sensory sensitivity–auditory. The sum of the modality scores for each of the four quadrants was used as the sub-score. The sum of the sub-scores was calculated, and the higher the sum of the sub-scores, the greater the quadrant tendency for modality-specific sensory processing. The information for each sub-score (mean, SD, range, and possible score) for the participants is shown in Table 2.

### Correlations between sub-scores of AASP and diffusion parameters in the caudate nucleus

**Fig 2:**
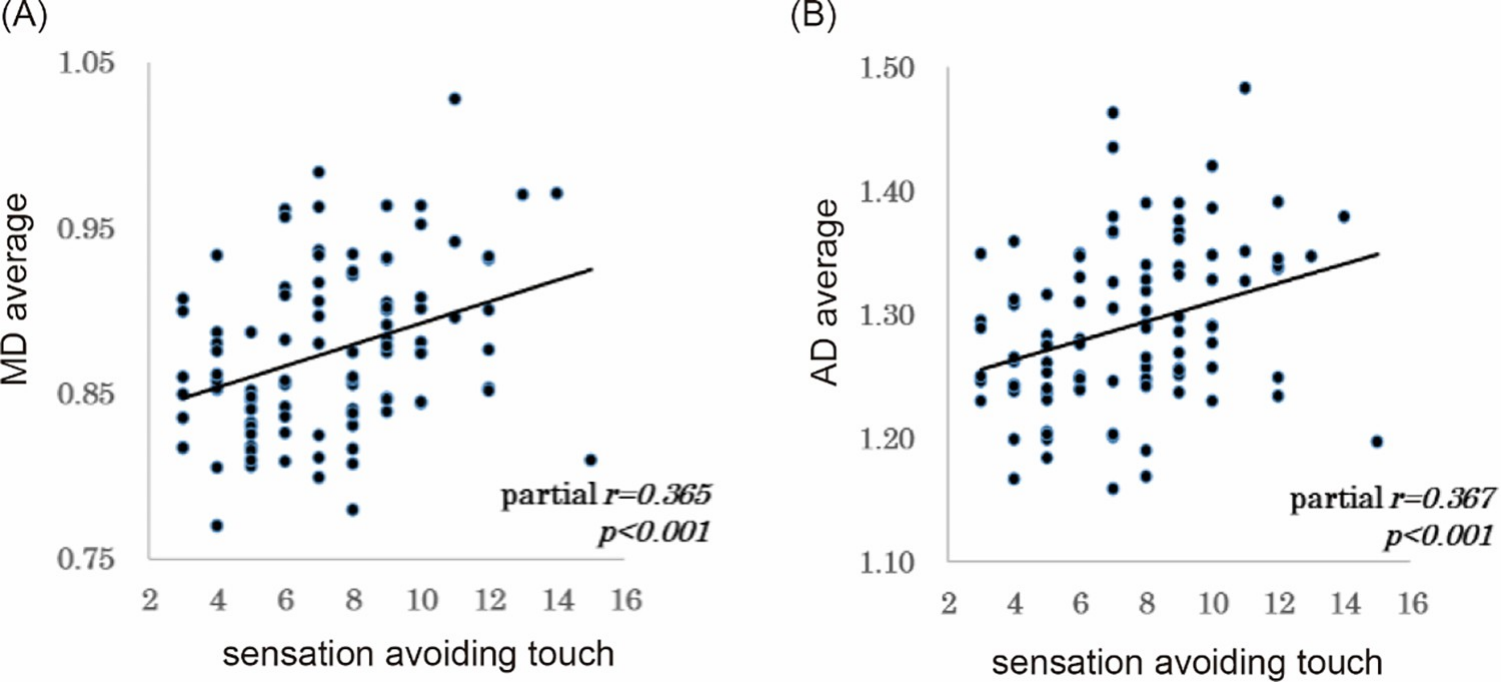
Correlation between the white matter fiber of the right caudate and AASP sub-score. A shows the correlations between MD of the white matter in the right caudate and the sensation avoiding touch score in AASP score (partial r = 0.365, p<0.001). B shows the correlations between AD of the white matter in the right caudate and the sensation avoiding touch score in AASP score (partial r = 0.367, p<0.001). B shows the correlations between MD of the white matter in the right caudate and the sensation avoiding touch score in AASP score (partial r = 0.365, p<0.001). AD, axonal diffusivity; MD, mean diffusivity; AASP, Adolescent/Adult Sensory Profile.

To examine the relation between the diffusion characteristics of the white matter microstructure in the bilateral caudate nuclei and sensory processing, we performed a series of partial correlation analyses of diffusion parameters and AASP sub-scores in the bilateral caudate nuclei with age and sex as control variables. While no significant correlation appeared between AASP scores and the diffusion parameters of left caudate nucleus, we found significant correlations for the right caudate nucleus as follows: correlation between AD and sensory avoidance-touch scores of AASP (partial r = 0.367, p<0.001) and correlation between MD and AASP sensory avoidance-touch scores (partial r = 0.365, p<0.001). The results of the correlation analysis are presented in Table 3 and Fig 2.

## Discussion

In the present study, we identified a positive correlation between two diffusion parameters of white matter (i.e., MD, AD) in the right caudate nucleus and AASP scores (i.e., touch and sensory avoiding touch). Converging evidence from animal and human studies indicates that the caudate nucleus is the critical sub-brain region that receives sensory stimulus information arriving at the basal ganglia [28–30]. One of the white matter pathways, the superior occipitofrontal fasciculus, connects the caudate nucleus with the frontal and parietal lobes [31] and is also widely involved in sensory-motor coordination, including response selection [32] and sensory cognition [33]. In addition, the caudate nucleus is embedded in different cortical-striatal loops, suggesting that the functional aspects of stimulation, action, and reward are different between these regions [34]. Taken together, our results provide substantial evidence that the white matter of the caudate nucleus plays a key role in sensory processing.

In an animal study with cats in which the caudate nucleus was removed, there were reports of loss of motivation, obsessive compulsive behavior, cognitive deficits, stimulus-obsessed behavior, and hyperactivity [35]. Furthermore, activation in the right caudate was correlated with questionnaire scores that quantified the intensity of romantic passion in functional MRI studies of individuals engaged in intense romantic love [36]. In these studies, the specific function of the caudate nucleus was defined as the control of approach-obsession behaviors, ranging from a simple approach to tactile sensory touch [37]. In terms of diseases, the caudate nucleus is also known to be a causative site for neurodegenerative diseases, such as autism spectrum disorder (ASD) and obsessive-compulsive disorder (OCD), and various mental disorders. For example, in an analysis of adults and children with ASD, a positive correlation exists between caudate nucleus volume and repetitive behaviors [38–41]. Patients with OCD are hypothesized to have abnormalities in the cortico-striatal-thalamic-cortical circuit, which runs from the cerebral cortex through the striatum of the basal ganglia, through the thalamus, and back to the cerebral cortex [42].

Our findings show a strong correlation between white matter microstructure in the caudate and sensory avoidance based on the AASP tactile sensory measure. Sensory processing-related items on the AASP measure responses to tactile stimuli on the skin. For example, AASP items related to tactile sensory avoidance include the following: “I try to avoid getting my hands dirty or wear gloves,” “I am more likely to move away if someone gets too close,” and “I don’t like people standing too close to me, so I try not to stand in lines or too close to people.” Tactile sensory avoidance is highly sensitive to touch and shows positive behavioral responses, such as avoiding touching others. A previous study has indicated that increasing tactile sensation of the hand was associated with an increased metabolic signal [43]. Moreover, an animal study suggested that nerve fibers in the caudate nucleus fire when an electrical stimulus is anticipated on the skin [44]. Taken together, our findings suggest that the structure and function of the caudate are closely associated with tactile responses.

Studies using DTI imaging have demonstrated an association between increased MD and clinical symptoms of sensory processing in patients with depression [45]. MD could be considered as a marker of the overall displacement of water molecules and the increased values are hypothesized to reflect degeneration of cellular membranes or inflammation [46]. The strong positive correlation between the MD of the caudate and tactile sensation or tactile sensory avoidance suggests the higher values of tactile sensation or tactile sensory avoidance are associated with a loss of integration in the white matter microstructure of caudate. Various speculations have been made on the relationship between AD and changes in nerve fibers, such as decrease in AD is suggested to be associated with axonal damage [25, 47–49]. On the other hand increased AD has been associated with axonal injury or damage that causes a decrease in axonal density or caliber, finally resulting in an increase in the extra-axonal space allowing water molecules to move faster [50]. Although we cannot determine the actual change of the nerve fiber in the present study, the strong positive correlation between the AD of the caudate and tactile sensation or tactile sensory avoidance suggests tactile sensation or tactile sensory avoidance is associated with changes in white matter structure.

In future studies, because we analyzed a set of white matter structures in the present study, a detailed analysis of each white matter fiber projecting to the caudate might be conducted. Moreover, analysis of the white matter structure of the caudate should be performed not only in healthy individuals but also in individuals with diseases that are hypothesized to involve the sensory processing mechanism of tactile perception controlled by the caudate, such as ASD.

Also, as mentioned above, the AASP has some limitations in that it is a self-report questionnaire. In the future, it is necessary to examine the correlation with the caudate nucleus through a paradigm that can be more objectively evaluated sensory processing. For instance, the pre-purse inhibition (PPI) paradigm measures the inhibition of startling sensory stimuli (“pulse”) preceded by weak stimuli (“prepulse”), and can quantitatively assess processing of sensory stimuli [51, 52]. The PPI deficit has been consistently reported in various disorders, including ASD and OCD [53, 54]. The PPI deficit may also be associated with lower caudate BOLD activation [55] and lesions of the dorsal straitum [56]. To clarity the role of the caudate nucleus in tactile sensory processing, it needs to be replicated with more various paradigms.

## Supporting information

S1 Table Demography data and caudate track information. (DOCX)

S2 Table Sensory profile information. (DOCX)

S3 Table Sub-score information of sensory profile. (DOCX)

## References

[1] Kilroy E, Aziz-Zadeh L, Cermak S. Ayres theories of autism and sensory integration revisited: What contemporary neuroscience has to say. Brain Sciences. Brain Sci; 2019. doi: 10.3390/brainsci9030068 30901886 PMC6468444

[2] Thye MD, Bednarz HM, Herringshaw AJ, Sartin EB, Kana RK. The impact of atypical sensory processing on social impairments in autism spectrum disorder. Developmental Cognitive Neuroscience. Elsevier; 2018. pp. 151–167. doi: 10.1016/j.dcn.2017.04.010 28545994 PMC6987885

[3] Miller LJ, Schoen SA, James K, Schaaf RC. Lessons learned: A pilot study on occupational therapy effectiveness for children with sensory modulation disorder. American Journal of Occupational Therapy. 2007;61: 161–169. doi: 10.5014/ajot.61.2.161 17436838

[4] Ben-Sasson A, Cermak SA, Orsmond GI, Tager-Flusberg H, Kadlec MB, Carter AS. Sensory clusters of toddlers with autism spectrum disorders: Differences in affective symptoms. J Child Psychol Psychiatry. 2008;49: 817–825. doi: 10.1111/j.1469-7610.2008.01899.x 18498344

[5] Jasmin E, Couture M, McKinley P, Reid G, Fombonne E, Gisel E. Sensori-motor and daily living skills of preschool children with autism spectrum disorders. J Autism Dev Disord. 2009;39: 231–241. doi: 10.1007/s10803-008-0617-z 18629623

[6] Dunn W. The impact of sensory processing abilities on the daily lives of young children and their families: A conceptual model. Infants Young Child. 1997;9: 23–35. doi: 10.1097/00001163-199704000-00005

[7] Metz AE, Boling D, Devore A, Holladay H, Liao JF, Vlutch K Vander. Dunn’s model of sensory processing: An investigation of the axes of the four-quadrant model in healthy adults running head: Dunn’s model of sensory processing in healthy adults. Brain Sci. 2019;9: 35. doi: 10.3390/brainsci9020035 30736461 PMC6406387

[8] Brown C, Tollefson N, Dunn W, Cromwell R, Filion D. The adult sensory profile: Measuring patterns of sensory processing. American Journal of Occupational Therapy. 2001;55: 75–82. doi: 10.5014/ajot.55.1.75 11216370

[9] Yoshimura S, Sato W, Kochiyama T, Uono S, Sawada R, Kubota Y, et al. Gray matter volumes of early sensory regions are associated with individual differences in sensory processing. Hum Brain Mapp. 2017;38: 6206–6217. doi: 10.1002/hbm.23822 28940867 PMC6867006

[10] Huang AX, Hughes TL, Sutton LR, Lawrence M, Chen X, Ji Z, et al. Understanding the self in individuals with Autism Spectrum Disorders (ASD): A review of literature. Frontiers in Psychology. 2017. pp. 1–8. doi: 10.3389/fpsyg.2017.01422 28878717 PMC5572253

[11] Zou L, Ding G, Abutalebi J, Shu H, Peng D. Structural plasticity of the left caudate in bimodal bilinguals. Cortex. 2012;48: 1197–1206. doi: 10.1016/j.cortex.2011.05.022 21741636

[12] Stoffers D, Altena E, Van Der Werf YD, Sanz-Arigita EJ, Voorn TA, Astill RG, et al. The caudate: A key node in the neuronal network imbalance of insomnia? Brain. 2014;137: 610–620. doi: 10.1093/brain/awt329 24285642 PMC3914473

[13] Seghatoleslam M, Ghadiri MK, Ghaffarian N, Speckmann EJ, Gorji A. Cortical spreading depression modulates the caudate nucleus activity. Neuroscience. 2014;267: 83–90. doi: 10.1016/j.neuroscience.2014.02.029 24613721

[14] Wunderlich AP, Klug R, Stuber G, Landwehrmeyer B, Weber F, Freund W. Caudate Nucleus and Insular Activation During a Pain Suppression Paradigm Comparing Thermal and Electrical Stimulation. Open Neuroimag J. 2011;5: 1–8. doi: 10.2174/1874440001105010001 21643502 PMC3106353

[15] Owen JP, Marco EJ, Desai S, Fourie E, Harris J, Hill SS, et al. Abnormal white matter microstructure in children with sensory processing disorders. Neuroimage Clin. 2013;2: 844–853. doi: 10.1016/j.nicl.2013.06.009 24179836 PMC3778265

[16] Jueptner M, Weiller C. A review of differences between basal ganglia and cerebellar control of movements as revealed by functional imaging studies. Brain. 1998;121: 1437–1449. doi: 10.1093/brain/121.8.1437 9712006

[17] Hickie IB, Naismith SL, Ward PB, Little CL, Pearson M, Scott EM, et al. Psychomotor slowing in older patients with major depression: Relationships with blood flow in the caudate nucleus and white matter lesions. Psychiatry Res Neuroimaging. 2007;155: 211–220. doi: 10.1016/j.pscychresns.2007.01.006 17574392

[18] Winklewski PJ, Sabisz A, Naumczyk P, Jodzio K, Szurowska E, Szarmach A. Understanding the physiopathology behind axial and radial diffusivity changes-what do we Know? Frontiers in Neurology. Front Neurol; 2018. doi: 10.3389/fneur.2018.00092 29535676 PMC5835085

[19] Madden DJ, Bennett IJ, Burzynska A, Potter GG, kuei Chen N, Song AW. Diffusion tensor imaging of cerebral white matter integrity in cognitive aging. Biochimica et Biophysica Acta—Molecular Basis of Disease. 2012. pp. 386–400. doi: 10.1016/j.bbadis.2011.08.003 21871957 PMC3241892

[20] Chang YS, Gratiot M, Owen JP, Brandes-Aitken A, Desai SS, Hill SS, et al. White matter microstructure is associated with auditory and tactile processing in children with and without sensory processing disorder. Front Neuroanat. 2016;9. doi: 10.3389/fnana.2015.00169 26858611 PMC4726807

[21] Duncan L, Georgiades K, Wang L, Van Lieshout RJ, MacMillan HL, Ferro MA, et al. Psychometric Evaluation of the Mini International Neuropsychiatric Interview for Children and Adolescents (MINI-KID). Psychol Assess. 2018;30: 916–928. doi: 10.1037/pas0000541 29199837

[22] Catana Brown; Winnie Dunn. The adolescent/adult sensory profile: user’s mannual. San Antonio, TX: The Psychological Corporation; 2002.

[23] Jung M, Mody M, Fujioka T, Kimura Y, Okazawa H, Kosaka H. Sex differences in white matter pathways related to language ability. Front Neurosci. 2019;13. doi: 10.3389/fnins.2019.00898 31555075 PMC6723765

[24] Backhausen LL, Herting MM, Buse J, Roessner V, Smolka MN, Vetter NC. Quality control of structural MRI images applied using FreeSurfer-a hands-on workflow to rate motion artifacts. Front Neurosci. 2016;10: 558. doi: 10.3389/fnins.2016.00558 27999528 PMC5138230

[25] Jung M, Mody M, Fujioka T, Kimura Y, Okazawa H, Kosaka H. Sex Differences in White Matter Pathways Related to Language Ability. Front Neurosci. 2019;13: 898. doi: 10.3389/fnins.2019.00898 31555075 PMC6723765

[26] Yeh F. DSI Studio. 2021. doi: 10.5281/ZENODO.4978980

[27] Yoldemir B, Acar B, Firat Z, Kiliçkesmez Ö. SMT: A reliability based interactive DTI tractography algorithm. IEEE Trans Med Imaging. 2012;31: 1929–1940. doi: 10.1109/TMI.2012.2210052 22851254

[28] Herrero MT, Barcia C, Navarro JM. Functional anatomy of thalamus and basal ganglia. Child’s Nervous System. 2002. pp. 386–404. doi: 10.1007/s00381-002-0604-1 12192499

[29] Nagy AJ, Berényi A, Gulya K, Norita M, Benedek G, Nagy A. Direct projection from the visual associative cortex to the caudate nucleus in the feline brain. Neurosci Lett. 2011;503: 52–57. doi: 10.1016/j.neulet.2011.08.007 21864648

[30] Nagy A, Eördegh G, Paróczy Z, Márkus Z, Benedek G. Multisensory integration in the basal ganglia. European Journal of Neuroscience. 2006;24: 917–924. doi: 10.1111/j.1460-9568.2006.04942.x 16930419

[31] Wycoco V, Shroff M, Sudhakar S, Lee W. White Matter Anatomy. What the Radiologist Needs to Know. Neuroimaging Clinics of North America. 2013. pp. 197–216. doi: 10.1016/j.nic.2012.12.002 23608685

[32] Groenewegen HJ. The basal ganglia and motor control. Neural plasticity. Neural Plast; 2003. pp. 107–120. doi: 10.1155/NP.2003.107 14640312 PMC2565420

[33] Grahn JA, Parkinson JA, Owen AM. The cognitive functions of the caudate nucleus. Prog Neurobiol. 2008;86: 141–155. doi: 10.1016/j.pneurobio.2008.09.004 18824075

[34] Haruno M, Kawato M. Different neural correlates of reward expectation and reward expectation error in the putamen and caudate nucleus during stimulus-action-reward association learning. J Neurophysiol. 2006;95: 948–959. doi: 10.1152/jn.00382.2005 16192338

[35] Villablanca JR, Olmstead CE. The striatum: A fine tuner of the brain. Acta Neurobiol Exp (Wars). 1982;42: 227–299. 7164850

[36] Aron A, Fisher H, Mashek DJ, Strong G, Li H, Brown LL. Reward, motivation, and emotion systems associated with early-stage intense romantic love. J Neurophysiol. 2005;94: 327–337. doi: 10.1152/jn.00838.2004 15928068

[37] Villablanca JR. Why do we have a caudate nucleus? Acta Neurobiologiae Experimentalis. Acta Neurobiol Exp (Wars); 2010. pp. 95–105. 20407491 10.55782/ane-2010-1778

[38] Brambilla P, Hardan A, Ucelli Di Nemi S, Perez J, Soares JC, Barale F. Brain anatomy and development in autism: Review of structural MRI studies. Brain Research Bulletin. Brain Res Bull; 2003. pp. 557–569. doi: 10.1016/j.brainresbull.2003.06.001 14519452

[39] Hollander E, Anagnostou E, Chaplin W, Esposito K, Haznedar MM, Licalzi E, et al. Striatal volume on magnetic resonance imaging and repetitive behaviors in autism. Biol Psychiatry. 2005;58: 226–232. doi: 10.1016/j.biopsych.2005.03.040 15939406

[40] Rojas DC, Peterson E, Winterrowd E, Reite ML, Rogers SJ, Tregellas JR. Regional gray matter volumetric changes in autism associated with social and repetitive behavior symptoms. BMC Psychiatry. 2006;6. doi: 10.1186/1471-244X-6-56 17166273 PMC1770914

[41] Chen Y, Juhás M, Greenshaw AJ, Hu Q, Meng X, Cui H, et al. Abnormal resting-state functional connectivity of the left caudate nucleus in obsessive-compulsive disorder. Neurosci Lett. 2016;623: 57–62. doi: 10.1016/j.neulet.2016.04.030 27143323

[42] Dunlop K, Woodside B, Olmsted M, Colton P, Giacobbe P, Downar J. Reductions in Cortico-Striatal Hyperconnectivity Accompany Successful Treatment of Obsessive-Compulsive Disorder with Dorsomedial Prefrontal rTMS. Neuropsychopharmacology. 2016;41: 1395–1403. doi: 10.1038/npp.2015.292 26440813 PMC4793124

[43] Weder BJ, Leenders KL, Vontobel P, Nienhusmeier M, Keel A, Zaunbauer W, et al. Impaired somatosensory discrimination of shape in Parkinson’s disease: Association with caudate nucleus dopaminergic function. Hum Brain Mapp. 1999;8: 1–12. 10432178 10.1002/(SICI)1097-0193(1999)8:1<1::AID-HBM1>3.0.CO;2-E PMC6873336

[44] Koyama T, Kato K, Mikami A. During pain-avoidance neurons activated in the macaque anterior cingulate and caudate. Neurosci Lett. 2000;283: 17–20. doi: 10.1016/s0304-3940(00)00894-6 10729623

[45] Spalletta G, Piras F, Caltagirone C, Fagioli S. Hippocampal multimodal structural changes and subclinical depression in healthy individuals. J Affect Disord. 2014;152–154: 105–112. doi: 10.1016/j.jad.2013.05.068 23800444

[46] Yrondi A, Nemmi F, Billoux S, Giron A, Sporer M, Taib S, et al. Significant decrease in hippocampus and amygdala mean diffusivity in treatment-resistant depression patients who respond to electroconvulsive therapy. Front Psychiatry. 2019;10. doi: 10.3389/fpsyt.2019.00694 31607967 PMC6761799

[47] Budde MD, Xie M, Cross AH, Song SK. Axial diffusivity is the primary correlate of axonal injury in the experimental autoimmune encephalomyelitis spinal cord: A quantitative pixelwise analysis. Journal of Neuroscience. 2009;29: 2805–2813. doi: 10.1523/JNEUROSCI.4605-08.2009 19261876 PMC2673458

[48] Curran KM, Emsell L, Leemans A. Quantitative DTI measures. Diffusion Tensor Imaging: A Practical Handbook. Springer, New York, NY; 2016. pp. 65–87. doi: 10.1007/978-1-4939-3118-7_5

[49] Song S-K, Yoshino J, Le TQ, Lin S-J, Sun S-W, Cross AH, et al. Demyelination increases radial diffusivity in corpus callosum of mouse brain. Neuroimage. 2005;26: 132–140. doi: 10.1016/j.neuroimage.2005.01.028 15862213

[50] Papadelis C, Butler EE, Rubenstein M, Sun L, Zollei L, Nimec D, et al. Reorganization of the somatosensory cortex in hemiplegic cerebral palsy associated with impaired sensory tracts. Neuroimage Clin. 2018;17: 198–212. doi: 10.1016/j.nicl.2017.10.021 29159037 PMC5683344

[51] Kumari V, Gray JA, Geyer MA, Ffytche D, Soni W, Mitterschiffthaler MT, et al. Neural correlates of tactile prepulse inhibition: A functional MRI study in normal and schizophrenic subjects. Psychiatry Res Neuroimaging. 2003;122: 99–113. doi: 10.1016/s0925-4927(02)00123-3 12714174

[52] Swerdlow NR, Karban B, Ploum Y, Sharp R, Geyer MA, Eastvold A. Tactile Prepuff Inhibition of Startle in Children with Tourette’s Syndrome: In Search of an “fMRI-Friendly” Startle Paradigm. 2001. doi: 10.1016/s0006-3223(01)01164-7 11690592

[53] Hoenig K, Hochrein A, Quednow BB, Maier W, Wagner M. Impaired prepulse inhibition of acoustic startle in obsessive-compulsive disorder. Biol Psychiatry. 2005;57: 1153–1158. doi: 10.1016/j.biopsych.2005.01.040 15866555

[54] Madsen GF, Bilenberg N, Cantio C, Oranje B. Increased prepulse inhibition and sensitization of the startle reflex in autistic children. Autism Research. 2014;7: 94–103. doi: 10.1002/aur.1337 24124111

[55] Hazlett EA, Buchsbaum MS, Zhang J, Newmark RE, Glanton CF, Zelmanova Y, et al. Frontal-striatal-thalamic mediodorsal nucleus dysfunction in schizophrenia-spectrum patients during sensorimotor gating. Neuroimage. 2008;42: 1164–1177. doi: 10.1016/j.neuroimage.2008.05.039 18588988 PMC2548278

[56] Baldan Ramsey LC, Xu M, Wood N, Pittenger C. Lesions of the dorsomedial striatum disrupt prepulse inhibition. Neuroscience. 2011;180: 222–228. doi: 10.1016/j.neuroscience.2011.01.041 21315809 PMC3070827

