# Supplementary material for: Region-based analysis of sensory processing using diffusion tensor imaging
Source: PLoS One. 2023 Apr 10;18(4):e0284250. doi: 10.1371/journal.pone.0284250 (PMC10085014; doi:10.1371/journal.pone.0284250)
Supplement: S1 Table — (DOCX) [file pone.0284250.s001.docx]

Supplementary table 1. Demography data and Caudate track information

| Demographic data | | | | Caudate_L | | | | | Caudate_R | | | | |
| --- | --- | --- | --- | --- | --- | --- | --- | --- | --- | --- | --- | --- | --- |
| ID | Age | Sex | BMI | number  of  tracts | fa mean | md mean | ad mean | rd mean | number  of  tracts | fa mean | md mean | ad mean | rd mean |
| JP_001 | 23 | 1 | 19.16 | 117 | 0.3777 | 1.06 | 1.4853 | 0.8473 | 96 | 0.4031 | 0.9637 | 1.3904 | 0.7504 |
| JP_002 | 25 | 1 | 19.05 | 195 | 0.392 | 0.9727 | 1.3847 | 0.7667 | 171 | 0.4025 | 0.8808 | 1.2778 | 0.6823 |
| JP_003 | 22 | 1 | 19.05 | 247 | 0.4054 | 0.9217 | 1.335 | 0.715 | 168 | 0.3606 | 0.8866 | 1.2383 | 0.7108 |
| JP_004 | 30 | 1 | 19.61 | 151 | 0.4215 | 0.9288 | 1.3617 | 0.7123 | 150 | 0.4148 | 0.8113 | 1.2013 | 0.6162 |
| JP_005 | 22 | 2 | 22.6 | 56 | 0.383 | 1.0514 | 1.4757 | 0.8392 | 102 | 0.3616 | 0.9701 | 1.3472 | 0.7815 |
| JP_006 | 30 | 2 | 19.11 | 101 | 0.3584 | 1.1912 | 1.6107 | 0.9814 | 214 | 0.4142 | 0.8514 | 1.2613 | 0.6465 |
| JP_007 | 31 | 1 | 21.47 | 366 | 0.3915 | 0.9995 | 1.4242 | 0.7871 | 336 | 0.3896 | 0.9637 | 1.3861 | 0.7526 |
| JP_008 | 25 | 2 | 18.97 | 253 | 0.3638 | 1.1708 | 1.6052 | 0.9536 | 189 | 0.3834 | 0.9708 | 1.3789 | 0.7667 |
| JP_009 | 20 | 2 | 20.81 | 299 | 0.3993 | 0.9987 | 1.4297 | 0.7832 | 487 | 0.3995 | 0.8822 | 1.2806 | 0.683 |
| JP_010 | 20 | 1 | 19.92 | 253 | 0.4169 | 1.0014 | 1.4672 | 0.7685 | 255 | 0.4211 | 0.9006 | 1.3398 | 0.681 |
| JP_011 | 23 | 2 | 19.98 | 406 | 0.4408 | 0.9665 | 1.4355 | 0.7321 | 258 | 0.4513 | 0.8494 | 1.2949 | 0.6267 |
| JP_012 | 21 | 1 | 22.57 | 309 | 0.4597 | 0.8333 | 1.2792 | 0.6103 | 426 | 0.4582 | 0.8304 | 1.2928 | 0.5992 |
| JP_013 | 22 | 2 | 21.91 | 336 | 0.3725 | 1.0212 | 1.4254 | 0.8191 | 291 | 0.375 | 0.8828 | 1.2565 | 0.696 |
| JP_014 | 22 | 1 | 20.08 | 295 | 0.4384 | 0.9564 | 1.4249 | 0.7222 | 285 | 0.4094 | 0.8967 | 1.3259 | 0.6821 |
| JP_015 | 21 | 1 | 25.01 | 422 | 0.4287 | 0.9413 | 1.386 | 0.7189 | 307 | 0.4324 | 0.9009 | 1.3486 | 0.677 |
| JP_016 | 30 | 1 | 19.75 | 414 | 0.4582 | 0.9369 | 1.4288 | 0.691 | 507 | 0.4496 | 0.8524 | 1.3086 | 0.6243 |
| JP_017 | 24 | 2 | 22.94 | 254 | 0.419 | 0.9236 | 1.3632 | 0.7037 | 227 | 0.422 | 0.875 | 1.2983 | 0.6633 |
| JP_018 | 38 | 2 | 20.66 | 297 | 0.3888 | 1.0269 | 1.4408 | 0.8199 | 265 | 0.3941 | 0.8533 | 1.234 | 0.6629 |
| JP_019 | 22 | 1 | 18.38 | 369 | 0.4653 | 0.8857 | 1.3594 | 0.6488 | 358 | 0.4581 | 0.8558 | 1.3198 | 0.6238 |
| JP_020 | 22 | 1 | 24.98 | 262 | 0.4288 | 0.9245 | 1.3427 | 0.7154 | 292 | 0.4386 | 0.7701 | 1.1679 | 0.5712 |
| JP_021 | 31 | 1 | 27.1 | 330 | 0.4116 | 1.007 | 1.4577 | 0.7817 | 342 | 0.4188 | 0.931 | 1.3673 | 0.7129 |
| JP_022 | 22 | 1 | 23.88 | 301 | 0.406 | 0.9993 | 1.4457 | 0.7761 | 304 | 0.4071 | 0.8463 | 1.2536 | 0.6426 |
| JP_023 | 29 | 2 | 20.96 | 277 | 0.4169 | 1.0294 | 1.498 | 0.7951 | 188 | 0.3824 | 0.8999 | 1.2898 | 0.705 |
| JP_024 | 33 | 2 | 24.46 | 282 | 0.4105 | 0.9511 | 1.3827 | 0.7353 | 322 | 0.4009 | 0.8789 | 1.2862 | 0.6753 |
| JP_025 | 22 | 1 | 21.22 | 308 | 0.4403 | 0.9834 | 1.4398 | 0.7552 | 369 | 0.4304 | 0.8805 | 1.3119 | 0.6648 |
| JP_026 | 21 | 1 | 21.72 | 341 | 0.4073 | 0.9648 | 1.3935 | 0.7504 | 286 | 0.3928 | 0.8552 | 1.2478 | 0.6589 |
| JP_027 | 23 | 2 | 19.4 | 341 | 0.4089 | 1.0026 | 1.4481 | 0.7799 | 220 | 0.3948 | 0.8515 | 1.2498 | 0.6524 |
| JP_028 | 21 | 1 | 23.3 | 367 | 0.4323 | 0.9511 | 1.4045 | 0.7244 | 265 | 0.4475 | 0.8762 | 1.3374 | 0.6456 |
| JP_029 | 22 | 2 | 20.7 | 419 | 0.4138 | 0.9555 | 1.3944 | 0.736 | 209 | 0.4164 | 0.9317 | 1.3765 | 0.7092 |
| JP_030 | 25 | 1 | 22.98 | 302 | 0.4057 | 0.9238 | 1.3375 | 0.7169 | 249 | 0.4334 | 0.8263 | 1.2502 | 0.6143 |
| JP_031 | 23 | 1 | 18.56 | 415 | 0.4053 | 1.033 | 1.4823 | 0.8083 | 314 | 0.4214 | 0.9344 | 1.39 | 0.7065 |
| JP_032 | 22 | 2 | 20.32 | 253 | 0.4371 | 0.902 | 1.3443 | 0.6808 | 236 | 0.4468 | 0.8089 | 1.2407 | 0.593 |
| JP_033 | 24 | 1 | 21.2 | 375 | 0.43 | 0.9957 | 1.477 | 0.755 | 183 | 0.4107 | 0.8376 | 1.2575 | 0.6276 |
| JP_034 | 25 | 1 | 22.66 | 216 | 0.4238 | 1.0602 | 1.5412 | 0.8197 | 256 | 0.3775 | 0.9615 | 1.3492 | 0.7677 |
| JP_035 | 23 | 1 | 24.74 | 387 | 0.4534 | 0.9634 | 1.4737 | 0.7083 | 311 | 0.4608 | 0.9171 | 1.4352 | 0.6581 |
| JP_036 | 22 | 2 | 25.08 | 269 | 0.3888 | 1.0796 | 1.5324 | 0.8532 | 268 | 0.3683 | 0.9567 | 1.3472 | 0.7614 |
| JP_037 | 22 | 1 | 20.05 | 406 | 0.4296 | 0.9684 | 1.4293 | 0.738 | 221 | 0.4226 | 0.9364 | 1.3795 | 0.7149 |
| JP_038 | 22 | 1 | 23.57 | 80 | 0.4719 | 1.0226 | 1.5628 | 0.7526 | 200 | 0.4607 | 0.9624 | 1.4631 | 0.7121 |
| JP_039 | 21 | 2 | 21.36 | 215 | 0.4661 | 0.9017 | 1.3838 | 0.6607 | 296 | 0.4237 | 0.8349 | 1.246 | 0.6293 |
| JP_040 | 22 | 2 | 19.98 | 242 | 0.4415 | 0.9644 | 1.428 | 0.7325 | 222 | 0.4446 | 0.9046 | 1.3609 | 0.6764 |
| JP_041 | 23 | 1 | 29.75 | 361 | 0.4306 | 0.9406 | 1.3908 | 0.7156 | 206 | 0.4433 | 0.8161 | 1.2471 | 0.6006 |
| JP_042 | 22 | 2 | 22.43 | 242 | 0.4198 | 0.9447 | 1.3815 | 0.7263 | 234 | 0.4216 | 0.84 | 1.2483 | 0.6359 |
| JP_043 | 23 | 1 | 18.29 | 334 | 0.4057 | 1.0387 | 1.4889 | 0.8136 | 316 | 0.3947 | 0.9313 | 1.3392 | 0.7273 |
| JP_044 | 21 | 1 | 21.55 | 300 | 0.3863 | 1.0219 | 1.4366 | 0.8146 | 283 | 0.401 | 0.9212 | 1.3406 | 0.7115 |
| JP_045 | 21 | 2 | 19.56 | 317 | 0.4239 | 0.874 | 1.2976 | 0.6622 | 282 | 0.4164 | 0.875 | 1.3029 | 0.6611 |
| JP_046 | 22 | 2 | 25.97 | 262 | 0.4192 | 0.9459 | 1.4005 | 0.7185 | 278 | 0.3812 | 0.8579 | 1.2396 | 0.667 |
| JP_047 | 20 | 2 | 19.84 | 303 | 0.4306 | 1.0509 | 1.5227 | 0.815 | 256 | 0.4395 | 0.9072 | 1.3496 | 0.6859 |
| JP_048 | 22 | 2 | 22.77 | 253 | 0.3963 | 1.0244 | 1.4387 | 0.8172 | 350 | 0.4431 | 0.8402 | 1.2837 | 0.6184 |
| JP_049 | 22 | 2 | 22.6 | 398 | 0.4373 | 0.9049 | 1.3519 | 0.6814 | 197 | 0.4211 | 0.8472 | 1.2514 | 0.6451 |
| JP_050 | 36 | 1 | 23.72 | 382 | 0.448 | 0.8826 | 1.3274 | 0.6602 | 318 | 0.4311 | 0.8322 | 1.2368 | 0.6299 |
| JP_051 | 29 | 1 | 20.76 | 269 | 0.4454 | 0.9569 | 1.4301 | 0.7203 | 235 | 0.3959 | 0.8757 | 1.2624 | 0.6823 |
| JP_052 | 22 | 2 | 22.31 | 417 | 0.4113 | 0.9292 | 1.3414 | 0.7231 | 361 | 0.4217 | 0.8297 | 1.2311 | 0.629 |
| JP_053 | 22 | 1 | 18.38 | 271 | 0.4349 | 1.1119 | 1.6208 | 0.8574 | 310 | 0.441 | 0.9524 | 1.4207 | 0.7182 |
| JP_054 | 23 | 2 | 19.92 | 213 | 0.3996 | 1.0637 | 1.5004 | 0.8454 | 235 | 0.378 | 0.8803 | 1.246 | 0.6974 |
| JP_055 | 21 | 2 | 20.94 | 305 | 0.4801 | 0.9324 | 1.4585 | 0.6694 | 262 | 0.4239 | 0.8387 | 1.2555 | 0.6303 |
| JP_056 | 22 | 2 | 19.88 | 337 | 0.36 | 1.0618 | 1.4638 | 0.8609 | 182 | 0.3816 | 0.9235 | 1.3284 | 0.721 |
| JP_057 | 25 | 2 | 27.43 | 267 | 0.401 | 0.9525 | 1.3648 | 0.7464 | 208 | 0.4028 | 0.8177 | 1.1995 | 0.6269 |
| JP_058 | 32 | 2 | 20.03 | 398 | 0.3987 | 0.9769 | 1.4021 | 0.7642 | 251 | 0.4126 | 0.9335 | 1.3592 | 0.7206 |
| JP_059 | 19 | 2 | 23.53 | 196 | 0.4068 | 0.9723 | 1.3965 | 0.7602 | 266 | 0.4295 | 0.8406 | 1.2769 | 0.6225 |
| JP_060 | 22 | 1 | 20.43 | 307 | 0.422 | 1.0034 | 1.4719 | 0.7691 | 363 | 0.3862 | 0.906 | 1.3052 | 0.7064 |
| JP_061 | 26 | 2 | 23.62 | 234 | 0.4031 | 0.9385 | 1.3366 | 0.7395 | 256 | 0.4366 | 0.8171 | 1.2306 | 0.6104 |
| JP_062 | 29 | 2 | 17.69 | 309 | 0.4182 | 0.9568 | 1.4026 | 0.7339 | 368 | 0.4287 | 0.8958 | 1.3512 | 0.6681 |
| JP_063 | 20 | 1 | 18.91 | 342 | 0.4252 | 0.9826 | 1.4396 | 0.7541 | 349 | 0.4351 | 0.8866 | 1.3162 | 0.6718 |
| JP_064 | 37 | 2 | 19.78 | 201 | 0.4255 | 0.9484 | 1.3914 | 0.7269 | 227 | 0.4033 | 0.8531 | 1.2433 | 0.658 |
| JP_065 | 24 | 1 | 21.94 | 307 | 0.464 | 0.876 | 1.3397 | 0.6442 | 374 | 0.42 | 0.8253 | 1.2402 | 0.6178 |
| JP_066 | 21 | 2 | 20.81 | 351 | 0.4131 | 0.9352 | 1.3621 | 0.7218 | 530 | 0.4051 | 0.9078 | 1.3286 | 0.6974 |
| JP_067 | 32 | 2 | 21.33 | 336 | 0.4135 | 0.9471 | 1.3727 | 0.7343 | 254 | 0.4163 | 0.8574 | 1.2653 | 0.6535 |
| JP_068 | 20 | 2 | 18.29 | 242 | 0.4407 | 0.8595 | 1.2845 | 0.647 | 298 | 0.4367 | 0.8481 | 1.2753 | 0.6345 |
| JP_069 | 22 | 2 | 19.38 | 314 | 0.4045 | 1.013 | 1.4544 | 0.7923 | 243 | 0.4005 | 0.9143 | 1.3307 | 0.706 |
| JP_070 | 30 | 2 | 26.5 | 247 | 0.4207 | 1.0068 | 1.4767 | 0.7718 | 169 | 0.395 | 0.9327 | 1.3455 | 0.7263 |
| JP_071 | 24 | 2 | 24.24 | 287 | 0.4283 | 0.9606 | 1.4091 | 0.7364 | 271 | 0.4331 | 0.8587 | 1.2891 | 0.6434 |
| JP_072 | 39 | 2 | 27.89 | 192 | 0.4464 | 0.8705 | 1.3167 | 0.6474 | 256 | 0.3894 | 0.8245 | 1.2031 | 0.6352 |
| JP_073 | 21 | 1 | 18.81 | 288 | 0.3916 | 0.9199 | 1.3048 | 0.7275 | 250 | 0.4416 | 0.8419 | 1.2763 | 0.6246 |
| JP_074 | 25 | 1 | 23.14 | 336 | 0.4076 | 0.9381 | 1.3531 | 0.7306 | 292 | 0.4029 | 0.8153 | 1.2056 | 0.6202 |
| JP_075 | 22 | 2 | 20.08 | 202 | 0.4249 | 0.9406 | 1.3776 | 0.7221 | 289 | 0.4241 | 0.8356 | 1.2482 | 0.6293 |
| JP_076 | 43 | 2 | 20.07 | 259 | 0.4149 | 0.8649 | 1.2701 | 0.6623 | 232 | 0.3937 | 0.807 | 1.1694 | 0.6259 |
| JP_077 | 33 | 2 | 22.1 | 385 | 0.4346 | 0.9154 | 1.3654 | 0.6904 | 461 | 0.4394 | 0.8445 | 1.2917 | 0.6209 |
| JP_078 | 22 | 1 | 30.35 | 105 | 0.4375 | 1.0456 | 1.5336 | 0.8016 | 225 | 0.4617 | 0.9006 | 1.3915 | 0.6552 |
| JP_079 | 42 | 2 | 19.47 | 262 | 0.4271 | 0.8744 | 1.282 | 0.6706 | 234 | 0.4234 | 0.8053 | 1.1991 | 0.6084 |
| JP_080 | 42 | 1 | 24.76 | 248 | 0.4066 | 0.9451 | 1.3656 | 0.7349 | 165 | 0.3821 | 0.8915 | 1.2698 | 0.7023 |
| JP_081 | 30 | 1 | 18.04 | 299 | 0.3866 | 0.8942 | 1.2706 | 0.7059 | 214 | 0.3651 | 0.875 | 1.2302 | 0.6975 |
| JP_082 | 31 | 1 | 24.34 | 501 | 0.4334 | 0.9989 | 1.4791 | 0.7588 | 265 | 0.4049 | 1.0276 | 1.4831 | 0.7998 |
| JP_083 | 25 | 1 | 25.9 | 144 | 0.3939 | 1.0488 | 1.4864 | 0.83 | 286 | 0.415 | 0.9336 | 1.3661 | 0.7173 |
| JP_084 | 25 | 2 | 19.96 | 527 | 0.4437 | 0.9712 | 1.4602 | 0.7267 | 259 | 0.4169 | 0.8446 | 1.2573 | 0.6383 |
| JP_085 | 27 | 2 | 22.48 | 181 | 0.416 | 0.9252 | 1.3636 | 0.706 | 92 | 0.356 | 0.9841 | 1.3673 | 0.7925 |
| JP_086 | 40 | 2 | 25.56 | 315 | 0.4549 | 0.8441 | 1.288 | 0.6222 | 235 | 0.4129 | 0.8056 | 1.1839 | 0.6164 |
| JP_087 | 40 | 2 | 22.64 | 404 | 0.3968 | 1.0161 | 1.4532 | 0.7975 | 338 | 0.4085 | 0.8596 | 1.2501 | 0.6643 |
| JP_088 | 36 | 2 | 21.83 | 337 | 0.4536 | 0.9568 | 1.4465 | 0.712 | 285 | 0.4222 | 0.9016 | 1.3328 | 0.6861 |
| JP_089 | 37 | 2 | 26.35 | 287 | 0.3909 | 0.8382 | 1.2073 | 0.6536 | 222 | 0.4374 | 0.7795 | 1.1905 | 0.574 |
| JP_090 | 39 | 2 | 18.07 | 227 | 0.3902 | 0.9827 | 1.3851 | 0.7816 | 199 | 0.4159 | 0.8383 | 1.2426 | 0.6361 |
| JP_091 | 37 | 2 | 20.08 | 309 | 0.4039 | 0.8616 | 1.2532 | 0.6658 | 334 | 0.3937 | 0.7992 | 1.1597 | 0.619 |
| JP_092 | 31 | 1 | 23.31 | 346 | 0.3995 | 0.9186 | 1.3151 | 0.7204 | 141 | 0.3927 | 0.8615 | 1.2419 | 0.6714 |
| JP_093 | 42 | 2 | 21.76 | 326 | 0.4013 | 0.9071 | 1.2919 | 0.7147 | 171 | 0.4236 | 0.8096 | 1.1976 | 0.6156 |
| JP_094 | 42 | 2 | 19 | 208 | 0.3799 | 0.9749 | 1.3701 | 0.7773 | 202 | 0.3767 | 0.9416 | 1.3271 | 0.7488 |
| JP_095 | 36 | 2 | 24.78 | 253 | 0.4071 | 0.8943 | 1.2955 | 0.6937 | 267 | 0.4098 | 0.8468 | 1.2369 | 0.6517 |
| JP_096 | 43 | 2 | 20.7 | 239 | 0.3899 | 0.9664 | 1.3569 | 0.7711 | 214 | 0.3995 | 0.8597 | 1.265 | 0.657 |
| JP_097 | 21 | 2 | 21.5 | 379 | 0.4016 | 0.9526 | 1.3751 | 0.7414 | 172 | 0.4171 | 0.8097 | 1.203 | 0.6131 |
| JP_098 | 32 | 1 | 23.1 | 439 | 0.4203 | 0.9772 | 1.4274 | 0.7521 | 253 | 0.4116 | 0.8743 | 1.2907 | 0.6661 |
| JP_099 | 43 | 2 | 19.36 | 396 | 0.397 | 0.9754 | 1.388 | 0.7691 | 302 | 0.4019 | 0.9092 | 1.3104 | 0.7086 |
