# Supplementary material for: Region-based analysis of sensory processing using diffusion tensor imaging
Source: PLoS One. 2023 Apr 10;18(4):e0284250. doi: 10.1371/journal.pone.0284250 (PMC10085014; doi:10.1371/journal.pone.0284250)
Supplement: S2 Table — (DOCX) [file pone.0284250.s002.docx]

Supplementary table 2. Sensory profile information

|  | Sensory profile | | | | | | | | | |
| --- | --- | --- | --- | --- | --- | --- | --- | --- | --- | --- |
| ID | Taste/Smell | Movement | Visual | Touch | Activity  level | Auditory | Low  registration | Sensation  seeking | Sensory  sensitivity | Sensation  avoiding |
| JP_001 | 21 | 20 | 25 | 38 | 28 | 34 | 36 | 45 | 46 | 39 |
| JP_002 | 18 | 16 | 25 | 28 | 27 | 21 | 30 | 24 | 40 | 41 |
| JP_003 | 14 | 15 | 20 | 23 | 18 | 15 | 15 | 36 | 27 | 27 |
| JP_004 | 14 | 14 | 19 | 24 | 27 | 24 | 21 | 36 | 31 | 34 |
| JP_005 | 21 | 24 | 28 | 48 | 29 | 26 | 32 | 53 | 45 | 46 |
| JP_006 | 16 | 18 | 16 | 28 | 28 | 16 | 26 | 42 | 29 | 25 |
| JP_007 | 15 | 24 | 30 | 36 | 33 | 39 | 35 | 50 | 49 | 43 |
| JP_008 | 17 | 14 | 25 | 36 | 29 | 16 | 22 | 30 | 44 | 41 |
| JP_009 | 19 | 16 | 22 | 28 | 34 | 19 | 36 | 37 | 31 | 34 |
| JP_010 | 25 | 22 | 29 | 48 | 25 | 25 | 40 | 44 | 51 | 39 |
| JP_011 | 11 | 17 | 34 | 19 | 29 | 33 | 37 | 29 | 43 | 34 |
| JP_012 | 19 | 21 | 24 | 34 | 34 | 35 | 37 | 50 | 40 | 40 |
| JP_013 | 12 | 19 | 21 | 34 | 26 | 20 | 29 | 38 | 35 | 30 |
| JP_014 | 12 | 12 | 20 | 22 | 18 | 22 | 20 | 25 | 30 | 31 |
| JP_015 | 14 | 21 | 21 | 27 | 32 | 24 | 30 | 37 | 30 | 42 |
| JP_016 | 15 | 15 | 21 | 18 | 32 | 27 | 30 | 40 | 27 | 31 |
| JP_017 | 20 | 21 | 24 | 35 | 33 | 28 | 38 | 36 | 46 | 41 |
| JP_018 | 22 | 16 | 25 | 37 | 25 | 18 | 24 | 37 | 41 | 41 |
| JP_019 | 21 | 16 | 24 | 36 | 32 | 25 | 39 | 42 | 37 | 36 |
| JP_020 | 12 | 17 | 18 | 26 | 20 | 18 | 20 | 45 | 27 | 19 |
| JP_021 | 8 | 16 | 26 | 35 | 24 | 21 | 26 | 39 | 29 | 36 |
| JP_022 | 18 | 18 | 25 | 24 | 27 | 23 | 29 | 44 | 29 | 33 |
| JP_023 | 16 | 13 | 20 | 25 | 26 | 19 | 27 | 42 | 27 | 23 |
| JP_024 | 17 | 21 | 24 | 30 | 24 | 26 | 23 | 40 | 36 | 43 |
| JP_025 | 17 | 22 | 20 | 27 | 30 | 18 | 34 | 40 | 31 | 29 |
| JP_026 | 9 | 13 | 23 | 24 | 23 | 21 | 18 | 28 | 33 | 34 |
| JP_027 | 19 | 21 | 27 | 29 | 25 | 23 | 21 | 38 | 41 | 44 |
| JP_028 | 19 | 15 | 24 | 30 | 36 | 26 | 34 | 32 | 38 | 46 |
| JP_029 | 14 | 16 | 25 | 36 | 26 | 17 | 18 | 46 | 36 | 34 |
| JP_030 | 21 | 18 | 25 | 30 | 30 | 24 | 37 | 48 | 31 | 32 |
| JP_031 | 18 | 13 | 23 | 33 | 30 | 25 | 32 | 41 | 38 | 31 |
| JP_032 | 16 | 14 | 18 | 26 | 19 | 12 | 17 | 35 | 25 | 28 |
| JP_033 | 15 | 20 | 23 | 29 | 23 | 25 | 23 | 38 | 39 | 35 |
| JP_034 | 10 | 20 | 17 | 23 | 26 | 21 | 26 | 31 | 31 | 29 |
| JP_035 | 16 | 21 | 33 | 34 | 28 | 33 | 29 | 48 | 47 | 41 |
| JP_036 | 17 | 15 | 23 | 34 | 29 | 19 | 26 | 39 | 40 | 32 |
| JP_037 | 25 | 24 | 27 | 23 | 31 | 22 | 30 | 46 | 38 | 38 |
| JP_038 | 14 | 15 | 21 | 29 | 27 | 18 | 19 | 48 | 28 | 29 |
| JP_039 | 15 | 14 | 19 | 15 | 18 | 24 | 28 | 32 | 22 | 23 |
| JP_040 | 13 | 16 | 26 | 38 | 32 | 28 | 34 | 47 | 39 | 33 |
| JP_041 | 19 | 18 | 18 | 26 | 29 | 19 | 27 | 37 | 28 | 37 |
| JP_042 | 17 | 21 | 20 | 31 | 28 | 29 | 24 | 36 | 43 | 43 |
| JP_043 | 21 | 18 | 19 | 34 | 37 | 26 | 30 | 41 | 41 | 43 |
| JP_044 | 16 | 20 | 27 | 23 | 21 | 19 | 19 | 45 | 31 | 31 |
| JP_045 | 18 | 19 | 24 | 29 | 30 | 24 | 31 | 48 | 33 | 32 |
| JP_046 | 15 | 9 | 19 | 25 | 24 | 21 | 30 | 28 | 25 | 30 |
| JP_047 | 8 | 16 | 15 | 17 | 20 | 16 | 23 | 29 | 22 | 18 |
| JP_048 | 17 | 23 | 26 | 30 | 26 | 26 | 35 | 47 | 33 | 33 |
| JP_049 | 23 | 15 | 25 | 38 | 24 | 22 | 36 | 49 | 31 | 31 |
| JP_050 | 16 | 14 | 19 | 25 | 24 | 22 | 23 | 37 | 28 | 32 |
| JP_051 | 15 | 18 | 20 | 25 | 19 | 18 | 18 | 50 | 24 | 23 |
| JP_052 | 13 | 16 | 26 | 29 | 29 | 31 | 32 | 49 | 33 | 30 |
| JP_053 | 14 | 16 | 22 | 35 | 29 | 29 | 27 | 33 | 42 | 43 |
| JP_054 | 20 | 20 | 25 | 29 | 24 | 24 | 29 | 46 | 34 | 33 |
| JP_055 | 20 | 10 | 30 | 31 | 27 | 28 | 35 | 28 | 38 | 45 |
| JP_056 | 16 | 15 | 26 | 34 | 24 | 27 | 36 | 34 | 36 | 36 |
| JP_057 | 20 | 17 | 19 | 20 | 22 | 23 | 20 | 42 | 33 | 26 |
| JP_058 | 13 | 11 | 11 | 22 | 14 | 15 | 19 | 23 | 21 | 23 |
| JP_059 | 18 | 15 | 20 | 24 | 22 | 15 | 24 | 33 | 30 | 27 |
| JP_060 | 15 | 17 | 17 | 30 | 26 | 18 | 24 | 44 | 28 | 27 |
| JP_061 | 14 | 12 | 22 | 21 | 20 | 20 | 18 | 52 | 19 | 20 |
| JP_062 | 16 | 22 | 31 | 29 | 29 | 22 | 25 | 41 | 36 | 47 |
| JP_063 | 16 | 17 | 21 | 24 | 30 | 21 | 27 | 53 | 21 | 28 |
| JP_064 | 20 | 22 | 19 | 30 | 22 | 17 | 20 | 40 | 37 | 33 |
| JP_065 | 20 | 18 | 17 | 22 | 31 | 18 | 26 | 51 | 26 | 23 |
| JP_066 | 24 | 15 | 32 | 39 | 25 | 24 | 31 | 43 | 46 | 39 |
| JP_067 | 13 | 15 | 25 | 21 | 27 | 18 | 27 | 38 | 28 | 26 |
| JP_068 | 15 | 26 | 32 | 32 | 37 | 31 | 48 | 47 | 43 | 35 |
| JP_069 | 17 | 19 | 29 | 32 | 25 | 22 | 27 | 43 | 34 | 40 |
| JP_070 | 19 | 14 | 26 | 41 | 32 | 22 | 37 | 43 | 32 | 42 |
| JP_071 | 13 | 16 | 23 | 31 | 20 | 17 | 17 | 46 | 29 | 28 |
| JP_072 | 14 | 12 | 19 | 27 | 26 | 16 | 27 | 29 | 33 | 25 |
| JP_073 | 17 | 15 | 18 | 23 | 23 | 18 | 25 | 31 | 26 | 32 |
| JP_074 | 15 | 20 | 27 | 24 | 27 | 22 | 26 | 45 | 33 | 31 |
| JP_075 | 13 | 15 | 19 | 26 | 24 | 18 | 25 | 36 | 30 | 24 |
| JP_076 | 15 | 13 | 22 | 28 | 29 | 18 | 26 | 41 | 32 | 26 |
| JP_077 | 23 | 22 | 25 | 34 | 25 | 33 | 35 | 44 | 44 | 39 |
| JP_078 | 17 | 13 | 35 | 31 | 22 | 25 | 25 | 35 | 34 | 49 |
| JP_079 | 18 | 21 | 20 | 19 | 23 | 20 | 27 | 47 | 25 | 22 |
| JP_080 | 20 | 17 | 18 | 28 | 20 | 19 | 18 | 41 | 30 | 33 |
| JP_081 | 15 | 11 | 17 | 28 | 26 | 17 | 25 | 35 | 27 | 27 |
| JP_082 | 13 | 16 | 21 | 27 | 27 | 16 | 21 | 43 | 24 | 32 |
| JP_083 | 17 | 17 | 16 | 26 | 24 | 19 | 24 | 43 | 25 | 27 |
| JP_084 | 21 | 14 | 23 | 25 | 33 | 31 | 31 | 37 | 36 | 43 |
| JP_085 | 12 | 20 | 20 | 24 | 26 | 21 | 22 | 40 | 29 | 32 |
| JP_086 | 20 | 14 | 17 | 22 | 17 | 16 | 19 | 36 | 27 | 24 |
| JP_087 | 14 | 19 | 18 | 24 | 20 | 16 | 28 | 39 | 27 | 17 |
| JP_088 | 19 | 16 | 23 | 36 | 31 | 27 | 31 | 43 | 38 | 40 |
| JP_089 | 17 | 18 | 23 | 34 | 27 | 19 | 31 | 37 | 39 | 31 |
| JP_090 | 24 | 20 | 24 | 31 | 32 | 25 | 34 | 48 | 41 | 33 |
| JP_091 | 16 | 19 | 29 | 25 | 19 | 32 | 32 | 30 | 34 | 44 |
| JP_092 | 20 | 17 | 19 | 22 | 23 | 22 | 25 | 38 | 27 | 33 |
| JP_093 | 15 | 10 | 23 | 37 | 26 | 26 | 20 | 34 | 38 | 45 |
| JP_094 | 20 | 16 | 25 | 38 | 27 | 21 | 26 | 45 | 34 | 42 |
| JP_095 | 21 | 23 | 30 | 40 | 31 | 28 | 32 | 52 | 47 | 42 |
| JP_096 | 14 | 25 | 32 | 39 | 26 | 23 | 33 | 40 | 48 | 38 |
| JP_097 | 16 | 16 | 25 | 32 | 31 | 27 | 36 | 46 | 36 | 29 |
| JP_098 | 17 | 25 | 26 | 33 | 35 | 31 | 45 | 46 | 39 | 37 |
| JP_099 | 22 | 21 | 33 | 34 | 25 | 18 | 26 | 55 | 39 | 33 |
