# Supplementary material for: Region-based analysis of sensory processing using diffusion tensor imaging
Source: PLoS One. 2023 Apr 10;18(4):e0284250. doi: 10.1371/journal.pone.0284250 (PMC10085014; doi:10.1371/journal.pone.0284250)
Supplement: S3 Table — (DOCX) [file pone.0284250.s003.docx]

Supplementary table 3. Sub-score information of sensory profile

|  | Taste/Smell | | | | Movement | | | | Visual | | | | Touch | | | | Activity level | | | | Auditory | | | |
| --- | --- | --- | --- | --- | --- | --- | --- | --- | --- | --- | --- | --- | --- | --- | --- | --- | --- | --- | --- | --- | --- | --- | --- | --- |
| ID | Low  registration | Sensation  seeking | Sensory  sensitivity | Sensation  avoiding | Low  registration | Sensation  seeking | Sensory  sensitivity | Sensation  avoiding | Low  registration | Sensation  seeking | Sensory  sensitivity | Sensation  avoiding | Low  registration | Sensation  seeking | Sensory  sensitivity | Sensation  avoiding | Low  registration | Sensation  seeking | Sensory  sensitivity | Sensation  avoiding | Low  registration | Sensation  seeking | Sensory  sensitivity | Sensation  avoiding |
| JP_001 | 6 | 8 | 4 | 3 | 4 | 8 | 7 | 1 | 4 | 4 | 8 | 9 | 6 | 10 | 13 | 9 | 7 | 7 | 5 | 9 | 9 | 8 | 9 | 8 |
| JP_002 | 4 | 4 | 4 | 6 | 2 | 6 | 6 | 2 | 4 | 3 | 9 | 9 | 3 | 5 | 10 | 10 | 10 | 3 | 5 | 9 | 7 | 3 | 6 | 5 |
| JP_003 | 2 | 5 | 3 | 4 | 2 | 8 | 4 | 1 | 2 | 6 | 6 | 6 | 3 | 8 | 8 | 4 | 3 | 6 | 1 | 8 | 3 | 3 | 5 | 4 |
| JP_004 | 2 | 7 | 3 | 2 | 3 | 7 | 3 | 1 | 2 | 5 | 3 | 9 | 3 | 4 | 10 | 7 | 5 | 8 | 4 | 10 | 6 | 5 | 8 | 5 |
| JP_005 | 4 | 10 | 3 | 4 | 4 | 10 | 7 | 3 | 3 | 7 | 8 | 10 | 7 | 13 | 15 | 13 | 8 | 7 | 4 | 10 | 6 | 6 | 8 | 6 |
| JP_006 | 4 | 8 | 1 | 3 | 3 | 10 | 4 | 1 | 2 | 6 | 4 | 4 | 4 | 6 | 13 | 5 | 9 | 7 | 3 | 9 | 4 | 5 | 4 | 3 |
| JP_007 | 2 | 6 | 3 | 4 | 5 | 10 | 8 | 1 | 4 | 6 | 13 | 7 | 5 | 12 | 9 | 10 | 12 | 7 | 5 | 9 | 7 | 9 | 11 | 12 |
| JP_008 | 2 | 6 | 5 | 4 | 3 | 2 | 8 | 1 | 6 | 2 | 10 | 7 | 3 | 7 | 12 | 14 | 5 | 8 | 5 | 11 | 3 | 5 | 4 | 4 |
| JP_009 | 2 | 3 | 5 | 9 | 5 | 7 | 3 | 1 | 6 | 7 | 4 | 5 | 7 | 8 | 7 | 6 | 10 | 9 | 5 | 10 | 6 | 3 | 7 | 3 |
| JP_010 | 4 | 10 | 5 | 6 | 5 | 4 | 12 | 1 | 7 | 4 | 9 | 9 | 12 | 11 | 16 | 9 | 5 | 9 | 2 | 9 | 7 | 6 | 7 | 5 |
| JP_011 | 2 | 5 | 1 | 3 | 4 | 5 | 7 | 1 | 8 | 3 | 11 | 12 | 3 | 4 | 9 | 3 | 9 | 5 | 5 | 10 | 11 | 7 | 10 | 5 |
| JP_012 | 5 | 8 | 1 | 5 | 4 | 9 | 7 | 1 | 3 | 6 | 9 | 6 | 6 | 10 | 10 | 8 | 9 | 9 | 4 | 12 | 10 | 8 | 9 | 8 |
| JP_013 | 2 | 5 | 3 | 2 | 5 | 8 | 5 | 1 | 5 | 6 | 4 | 6 | 4 | 8 | 13 | 9 | 8 | 8 | 3 | 7 | 5 | 3 | 7 | 5 |
| JP_014 | 2 | 6 | 1 | 3 | 2 | 4 | 5 | 1 | 3 | 4 | 7 | 6 | 3 | 4 | 8 | 7 | 5 | 4 | 2 | 7 | 5 | 3 | 7 | 7 |
| JP_015 | 4 | 5 | 1 | 4 | 4 | 7 | 8 | 2 | 2 | 5 | 5 | 9 | 6 | 6 | 5 | 10 | 9 | 8 | 4 | 11 | 5 | 6 | 7 | 6 |
| JP_016 | 2 | 10 | 1 | 2 | 3 | 6 | 5 | 1 | 3 | 6 | 4 | 8 | 4 | 5 | 5 | 4 | 10 | 8 | 5 | 9 | 8 | 5 | 7 | 7 |
| JP_017 | 5 | 5 | 4 | 6 | 5 | 6 | 9 | 1 | 6 | 3 | 9 | 6 | 5 | 9 | 12 | 9 | 7 | 10 | 4 | 12 | 10 | 3 | 8 | 7 |
| JP_018 | 2 | 6 | 4 | 10 | 3 | 6 | 6 | 1 | 4 | 7 | 9 | 5 | 5 | 8 | 12 | 12 | 7 | 8 | 3 | 7 | 3 | 2 | 7 | 6 |
| JP_019 | 5 | 9 | 2 | 5 | 4 | 6 | 5 | 1 | 3 | 4 | 8 | 9 | 7 | 10 | 11 | 8 | 11 | 8 | 4 | 9 | 9 | 5 | 7 | 4 |
| JP_020 | 2 | 5 | 3 | 2 | 3 | 10 | 3 | 1 | 3 | 5 | 5 | 5 | 3 | 10 | 9 | 4 | 5 | 9 | 2 | 4 | 4 | 6 | 5 | 3 |
| JP_021 | 2 | 3 | 1 | 2 | 2 | 10 | 3 | 1 | 5 | 6 | 6 | 9 | 9 | 7 | 10 | 9 | 4 | 9 | 4 | 7 | 4 | 4 | 5 | 8 |
| JP_022 | 4 | 7 | 3 | 4 | 3 | 10 | 4 | 1 | 4 | 6 | 6 | 9 | 5 | 7 | 7 | 5 | 8 | 8 | 3 | 8 | 5 | 6 | 6 | 6 |
| JP_023 | 3 | 10 | 1 | 2 | 3 | 5 | 4 | 1 | 3 | 5 | 5 | 7 | 5 | 9 | 8 | 3 | 7 | 10 | 3 | 6 | 6 | 3 | 6 | 4 |
| JP_024 | 3 | 3 | 4 | 7 | 3 | 9 | 8 | 1 | 3 | 6 | 8 | 7 | 5 | 10 | 6 | 9 | 5 | 8 | 2 | 9 | 4 | 4 | 8 | 10 |
| JP_025 | 5 | 7 | 2 | 3 | 5 | 8 | 6 | 3 | 4 | 4 | 6 | 6 | 6 | 7 | 10 | 4 | 9 | 9 | 3 | 9 | 5 | 5 | 4 | 4 |
| JP_026 | 2 | 3 | 2 | 2 | 2 | 6 | 4 | 1 | 3 | 3 | 9 | 8 | 3 | 7 | 8 | 6 | 4 | 5 | 3 | 11 | 4 | 4 | 7 | 6 |
| JP_027 | 3 | 6 | 3 | 7 | 2 | 10 | 8 | 1 | 3 | 6 | 10 | 8 | 3 | 6 | 8 | 12 | 4 | 7 | 4 | 10 | 6 | 3 | 8 | 6 |
| JP_028 | 3 | 8 | 1 | 7 | 3 | 5 | 6 | 1 | 3 | 4 | 8 | 9 | 3 | 6 | 9 | 12 | 12 | 6 | 5 | 13 | 10 | 3 | 9 | 4 |
| JP_029 | 2 | 4 | 3 | 5 | 2 | 9 | 4 | 1 | 3 | 7 | 7 | 8 | 3 | 10 | 14 | 9 | 4 | 11 | 4 | 7 | 4 | 5 | 4 | 4 |
| JP_030 | 6 | 6 | 4 | 5 | 4 | 10 | 3 | 1 | 5 | 5 | 7 | 8 | 8 | 9 | 7 | 6 | 8 | 10 | 4 | 8 | 6 | 8 | 6 | 4 |
| JP_031 | 3 | 9 | 2 | 4 | 3 | 4 | 5 | 1 | 4 | 7 | 6 | 6 | 3 | 8 | 14 | 8 | 10 | 8 | 4 | 8 | 9 | 5 | 7 | 4 |
| JP_032 | 2 | 6 | 1 | 7 | 2 | 8 | 3 | 1 | 3 | 6 | 5 | 4 | 3 | 7 | 10 | 6 | 4 | 6 | 2 | 7 | 3 | 2 | 4 | 3 |
| JP_033 | 2 | 4 | 4 | 5 | 5 | 9 | 5 | 1 | 3 | 7 | 6 | 7 | 3 | 7 | 11 | 8 | 5 | 6 | 4 | 8 | 5 | 5 | 9 | 6 |
| JP_034 | 2 | 4 | 1 | 3 | 2 | 7 | 10 | 1 | 3 | 3 | 5 | 6 | 3 | 8 | 6 | 6 | 9 | 5 | 3 | 9 | 7 | 4 | 6 | 4 |
| JP_035 | 3 | 7 | 3 | 3 | 3 | 10 | 7 | 1 | 3 | 7 | 12 | 11 | 7 | 7 | 13 | 7 | 7 | 9 | 3 | 9 | 6 | 8 | 9 | 10 |
| JP_036 | 2 | 8 | 2 | 5 | 2 | 4 | 8 | 1 | 3 | 5 | 7 | 8 | 6 | 10 | 12 | 6 | 9 | 8 | 5 | 7 | 4 | 4 | 6 | 5 |
| JP_037 | 5 | 10 | 5 | 5 | 5 | 10 | 7 | 2 | 3 | 8 | 9 | 7 | 3 | 5 | 8 | 7 | 7 | 9 | 3 | 12 | 7 | 4 | 6 | 5 |
| JP_038 | 3 | 7 | 1 | 3 | 2 | 9 | 3 | 1 | 2 | 6 | 6 | 7 | 3 | 8 | 11 | 7 | 6 | 11 | 3 | 7 | 3 | 7 | 4 | 4 |
| JP_039 | 5 | 6 | 1 | 3 | 4 | 5 | 4 | 1 | 2 | 7 | 5 | 5 | 5 | 3 | 4 | 3 | 6 | 6 | 1 | 5 | 6 | 5 | 7 | 6 |
| JP_040 | 3 | 7 | 1 | 2 | 4 | 5 | 6 | 1 | 6 | 6 | 6 | 8 | 6 | 9 | 14 | 9 | 8 | 10 | 4 | 10 | 7 | 10 | 8 | 3 |
| JP_041 | 4 | 6 | 2 | 7 | 2 | 9 | 6 | 1 | 4 | 4 | 3 | 7 | 5 | 5 | 8 | 8 | 8 | 8 | 3 | 10 | 4 | 5 | 6 | 4 |
| JP_042 | 2 | 6 | 3 | 6 | 3 | 6 | 9 | 3 | 2 | 4 | 7 | 7 | 5 | 8 | 10 | 8 | 6 | 9 | 3 | 10 | 6 | 3 | 11 | 9 |
| JP_043 | 4 | 7 | 5 | 5 | 2 | 8 | 7 | 1 | 2 | 4 | 5 | 8 | 4 | 7 | 11 | 12 | 10 | 9 | 5 | 13 | 8 | 6 | 8 | 4 |
| JP_044 | 4 | 6 | 1 | 5 | 2 | 10 | 7 | 1 | 4 | 10 | 6 | 7 | 3 | 3 | 9 | 8 | 3 | 10 | 1 | 7 | 3 | 6 | 7 | 3 |
| JP_045 | 4 | 5 | 4 | 5 | 4 | 8 | 6 | 1 | 3 | 8 | 6 | 7 | 4 | 7 | 10 | 8 | 10 | 10 | 3 | 7 | 6 | 10 | 4 | 4 |
| JP_046 | 4 | 5 | 1 | 5 | 2 | 3 | 3 | 1 | 3 | 4 | 6 | 6 | 6 | 8 | 5 | 6 | 6 | 4 | 5 | 9 | 9 | 4 | 5 | 3 |
| JP_047 | 2 | 3 | 1 | 2 | 2 | 9 | 4 | 1 | 4 | 2 | 4 | 5 | 3 | 7 | 4 | 3 | 6 | 6 | 4 | 4 | 6 | 2 | 5 | 3 |
| JP_048 | 3 | 8 | 2 | 4 | 5 | 9 | 6 | 3 | 4 | 7 | 6 | 9 | 7 | 11 | 7 | 5 | 8 | 8 | 3 | 7 | 8 | 4 | 9 | 5 |
| JP_049 | 7 | 10 | 1 | 5 | 3 | 5 | 6 | 1 | 4 | 10 | 4 | 7 | 9 | 9 | 11 | 9 | 7 | 8 | 3 | 6 | 6 | 7 | 6 | 3 |
| JP_050 | 2 | 7 | 2 | 5 | 2 | 8 | 3 | 1 | 2 | 5 | 5 | 7 | 5 | 5 | 10 | 5 | 8 | 6 | 2 | 8 | 4 | 6 | 6 | 6 |
| JP_051 | 2 | 9 | 1 | 3 | 2 | 10 | 5 | 1 | 2 | 7 | 4 | 7 | 4 | 10 | 7 | 4 | 4 | 8 | 2 | 5 | 4 | 6 | 5 | 3 |
| JP_052 | 2 | 6 | 3 | 2 | 3 | 8 | 3 | 2 | 6 | 7 | 8 | 5 | 4 | 14 | 6 | 5 | 9 | 7 | 4 | 9 | 8 | 7 | 9 | 7 |
| JP_053 | 3 | 5 | 2 | 4 | 4 | 4 | 7 | 1 | 3 | 3 | 8 | 8 | 4 | 7 | 14 | 10 | 6 | 9 | 4 | 10 | 7 | 5 | 7 | 10 |
| JP_054 | 4 | 9 | 3 | 4 | 5 | 8 | 6 | 1 | 3 | 7 | 8 | 7 | 5 | 9 | 8 | 7 | 6 | 6 | 3 | 9 | 6 | 7 | 6 | 5 |
| JP_055 | 4 | 8 | 1 | 7 | 4 | 2 | 3 | 1 | 5 | 6 | 11 | 8 | 5 | 6 | 11 | 9 | 8 | 3 | 4 | 12 | 9 | 3 | 8 | 8 |
| JP_056 | 5 | 7 | 2 | 2 | 2 | 4 | 8 | 1 | 5 | 6 | 6 | 9 | 8 | 8 | 10 | 8 | 9 | 6 | 2 | 7 | 7 | 3 | 8 | 9 |
| JP_057 | 2 | 10 | 5 | 3 | 2 | 8 | 6 | 1 | 2 | 6 | 6 | 5 | 3 | 5 | 7 | 5 | 6 | 7 | 3 | 6 | 5 | 6 | 6 | 6 |
| JP_058 | 2 | 5 | 1 | 5 | 2 | 3 | 5 | 1 | 2 | 2 | 3 | 4 | 6 | 7 | 5 | 4 | 3 | 4 | 2 | 5 | 4 | 2 | 5 | 4 |
| JP_059 | 3 | 6 | 3 | 6 | 2 | 6 | 6 | 1 | 4 | 5 | 6 | 5 | 3 | 7 | 9 | 5 | 7 | 6 | 2 | 7 | 5 | 3 | 4 | 3 |
| JP_060 | 2 | 7 | 2 | 4 | 2 | 10 | 4 | 1 | 4 | 5 | 5 | 3 | 4 | 10 | 9 | 7 | 7 | 8 | 3 | 8 | 5 | 4 | 5 | 4 |
| JP_061 | 2 | 9 | 1 | 2 | 2 | 6 | 3 | 1 | 2 | 10 | 4 | 6 | 3 | 9 | 6 | 3 | 4 | 10 | 1 | 5 | 5 | 8 | 4 | 3 |
| JP_062 | 2 | 6 | 5 | 3 | 3 | 10 | 6 | 3 | 5 | 5 | 10 | 11 | 3 | 7 | 8 | 11 | 8 | 7 | 2 | 12 | 4 | 6 | 5 | 7 |
| JP_063 | 2 | 10 | 1 | 3 | 3 | 10 | 3 | 1 | 2 | 8 | 4 | 7 | 4 | 8 | 7 | 5 | 9 | 10 | 2 | 9 | 7 | 7 | 4 | 3 |
| JP_064 | 2 | 8 | 4 | 6 | 4 | 5 | 10 | 3 | 2 | 5 | 5 | 7 | 5 | 10 | 11 | 4 | 4 | 6 | 3 | 9 | 3 | 6 | 4 | 4 |
| JP_065 | 2 | 11 | 3 | 4 | 3 | 9 | 5 | 1 | 4 | 4 | 6 | 3 | 3 | 10 | 4 | 5 | 9 | 10 | 5 | 7 | 5 | 7 | 3 | 3 |
| JP_066 | 3 | 9 | 5 | 7 | 2 | 6 | 6 | 1 | 6 | 8 | 10 | 8 | 7 | 10 | 12 | 10 | 7 | 5 | 4 | 9 | 6 | 5 | 9 | 4 |
| JP_067 | 3 | 7 | 1 | 2 | 4 | 4 | 6 | 1 | 4 | 7 | 7 | 7 | 4 | 7 | 6 | 4 | 7 | 8 | 4 | 8 | 5 | 5 | 4 | 4 |
| JP_068 | 3 | 6 | 4 | 2 | 5 | 8 | 10 | 3 | 7 | 6 | 10 | 9 | 7 | 12 | 8 | 5 | 15 | 9 | 2 | 11 | 11 | 6 | 9 | 5 |
| JP_069 | 2 | 6 | 3 | 6 | 4 | 9 | 5 | 1 | 5 | 7 | 8 | 9 | 7 | 11 | 8 | 6 | 4 | 7 | 2 | 12 | 5 | 3 | 8 | 6 |
| JP_070 | 4 | 7 | 1 | 7 | 5 | 3 | 5 | 1 | 6 | 6 | 8 | 6 | 6 | 12 | 11 | 12 | 10 | 9 | 2 | 11 | 6 | 6 | 5 | 5 |
| JP_071 | 2 | 8 | 1 | 2 | 2 | 10 | 3 | 1 | 3 | 7 | 5 | 8 | 4 | 6 | 13 | 8 | 3 | 9 | 2 | 6 | 3 | 6 | 5 | 3 |
| JP_072 | 2 | 9 | 1 | 2 | 4 | 2 | 5 | 1 | 4 | 3 | 9 | 3 | 3 | 6 | 11 | 7 | 9 | 7 | 1 | 9 | 5 | 2 | 6 | 3 |
| JP_073 | 4 | 5 | 3 | 5 | 2 | 8 | 4 | 1 | 2 | 4 | 5 | 7 | 4 | 6 | 7 | 6 | 6 | 6 | 2 | 9 | 7 | 2 | 5 | 4 |
| JP_074 | 3 | 7 | 2 | 3 | 3 | 9 | 7 | 1 | 3 | 8 | 6 | 10 | 4 | 5 | 10 | 5 | 7 | 8 | 4 | 8 | 6 | 8 | 4 | 4 |
| JP_075 | 2 | 6 | 3 | 2 | 3 | 6 | 5 | 1 | 3 | 6 | 4 | 6 | 4 | 7 | 9 | 6 | 7 | 8 | 3 | 6 | 6 | 3 | 6 | 3 |
| JP_076 | 2 | 9 | 1 | 3 | 2 | 2 | 8 | 1 | 3 | 7 | 8 | 4 | 6 | 8 | 6 | 8 | 7 | 11 | 4 | 7 | 6 | 4 | 5 | 3 |
| JP_077 | 5 | 11 | 2 | 5 | 6 | 7 | 8 | 1 | 3 | 7 | 8 | 7 | 6 | 7 | 11 | 10 | 7 | 6 | 4 | 8 | 8 | 6 | 11 | 8 |
| JP_078 | 4 | 6 | 1 | 6 | 3 | 4 | 5 | 1 | 5 | 5 | 12 | 13 | 4 | 8 | 7 | 12 | 4 | 7 | 1 | 10 | 5 | 5 | 8 | 7 |
| JP_079 | 4 | 9 | 2 | 3 | 4 | 10 | 6 | 1 | 3 | 8 | 4 | 5 | 4 | 5 | 6 | 4 | 6 | 8 | 3 | 6 | 6 | 7 | 4 | 3 |
| JP_080 | 2 | 7 | 5 | 6 | 2 | 10 | 4 | 1 | 2 | 8 | 4 | 4 | 4 | 7 | 8 | 9 | 3 | 7 | 2 | 8 | 5 | 2 | 7 | 5 |
| JP_081 | 2 | 8 | 2 | 3 | 2 | 5 | 3 | 1 | 3 | 4 | 5 | 5 | 4 | 5 | 9 | 10 | 9 | 10 | 2 | 5 | 5 | 3 | 6 | 3 |
| JP_082 | 2 | 7 | 2 | 2 | 2 | 8 | 5 | 1 | 4 | 6 | 6 | 5 | 5 | 6 | 5 | 11 | 4 | 11 | 2 | 10 | 4 | 5 | 4 | 3 |
| JP_083 | 3 | 9 | 2 | 3 | 3 | 8 | 5 | 1 | 2 | 6 | 4 | 4 | 5 | 7 | 7 | 7 | 5 | 8 | 3 | 8 | 6 | 5 | 4 | 4 |
| JP_084 | 4 | 7 | 4 | 6 | 3 | 5 | 5 | 1 | 4 | 5 | 6 | 8 | 4 | 4 | 7 | 10 | 8 | 10 | 5 | 10 | 8 | 6 | 9 | 8 |
| JP_085 | 3 | 5 | 1 | 3 | 4 | 6 | 9 | 1 | 2 | 4 | 8 | 6 | 5 | 7 | 5 | 7 | 3 | 10 | 3 | 10 | 5 | 8 | 3 | 5 |
| JP_086 | 2 | 8 | 3 | 7 | 3 | 4 | 6 | 1 | 2 | 5 | 5 | 5 | 4 | 8 | 5 | 5 | 3 | 7 | 4 | 3 | 5 | 4 | 4 | 3 |
| JP_087 | 2 | 8 | 1 | 3 | 4 | 8 | 6 | 1 | 4 | 5 | 6 | 3 | 9 | 7 | 5 | 3 | 4 | 8 | 4 | 4 | 5 | 3 | 5 | 3 |
| JP_088 | 3 | 8 | 2 | 6 | 3 | 5 | 7 | 1 | 3 | 5 | 7 | 8 | 6 | 11 | 10 | 9 | 9 | 9 | 3 | 10 | 7 | 5 | 9 | 6 |
| JP_089 | 4 | 6 | 2 | 5 | 4 | 6 | 7 | 1 | 4 | 5 | 9 | 5 | 5 | 10 | 11 | 8 | 8 | 7 | 4 | 8 | 6 | 3 | 6 | 4 |
| JP_090 | 4 | 12 | 3 | 5 | 4 | 7 | 8 | 1 | 5 | 8 | 7 | 4 | 7 | 6 | 10 | 8 | 6 | 10 | 5 | 11 | 8 | 5 | 8 | 4 |
| JP_091 | 3 | 6 | 1 | 6 | 6 | 6 | 6 | 1 | 5 | 5 | 8 | 11 | 6 | 4 | 8 | 7 | 5 | 4 | 2 | 8 | 7 | 5 | 9 | 11 |
| JP_092 | 3 | 9 | 3 | 5 | 4 | 6 | 4 | 3 | 4 | 4 | 5 | 6 | 3 | 8 | 7 | 4 | 6 | 7 | 2 | 8 | 5 | 4 | 6 | 7 |
| JP_093 | 3 | 8 | 1 | 3 | 2 | 2 | 5 | 1 | 3 | 2 | 9 | 9 | 3 | 7 | 12 | 15 | 6 | 6 | 3 | 11 | 3 | 9 | 8 | 6 |
| JP_094 | 3 | 9 | 3 | 5 | 4 | 6 | 5 | 1 | 3 | 4 | 7 | 11 | 6 | 11 | 10 | 11 | 7 | 9 | 3 | 8 | 3 | 6 | 6 | 6 |
| JP_095 | 2 | 12 | 1 | 6 | 4 | 8 | 8 | 3 | 4 | 8 | 10 | 8 | 4 | 10 | 17 | 9 | 10 | 9 | 3 | 9 | 8 | 5 | 8 | 7 |
| JP_096 | 2 | 5 | 3 | 4 | 6 | 4 | 11 | 4 | 5 | 7 | 11 | 9 | 8 | 10 | 13 | 8 | 6 | 6 | 5 | 9 | 6 | 8 | 5 | 4 |
| JP_097 | 3 | 7 | 3 | 3 | 5 | 2 | 8 | 1 | 5 | 5 | 7 | 8 | 6 | 11 | 10 | 5 | 9 | 12 | 3 | 7 | 8 | 9 | 5 | 5 |
| JP_098 | 5 | 6 | 1 | 5 | 6 | 10 | 8 | 1 | 5 | 8 | 8 | 5 | 5 | 7 | 11 | 10 | 12 | 10 | 4 | 9 | 12 | 5 | 7 | 7 |
| JP_099 | 5 | 10 | 2 | 5 | 4 | 8 | 8 | 1 | 4 | 8 | 13 | 8 | 7 | 12 | 9 | 6 | 3 | 10 | 2 | 10 | 3 | 7 | 5 | 3 |
